# Supplementary material for: ETV2 regulates PARP-1 binding protein to induce ER stress–mediated death in tuberin-deficient cells
Source: Life Sci Alliance. 2022 Feb 18;5(5):e202201369. doi: 10.26508/lsa.202201369 (PMC8860090; doi:10.26508/lsa.202201369)
Supplement: Supplementary file 1 [file LSA-2022-01369_SdataF2_F3_F4_F6_FS1_FS2_FS3_FS4_FS6.pdf]

FIGURE 2B

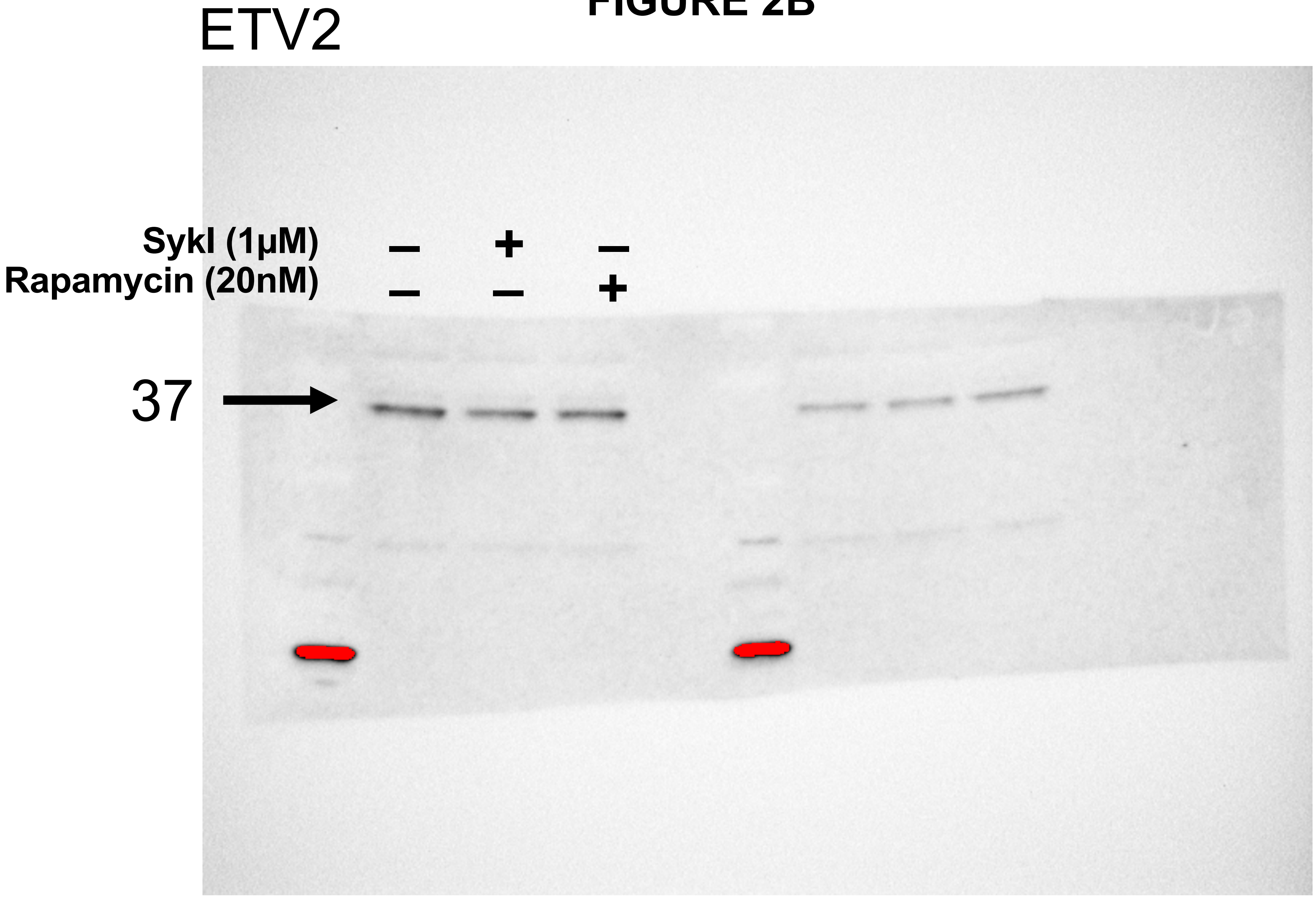

FIGURE 2B

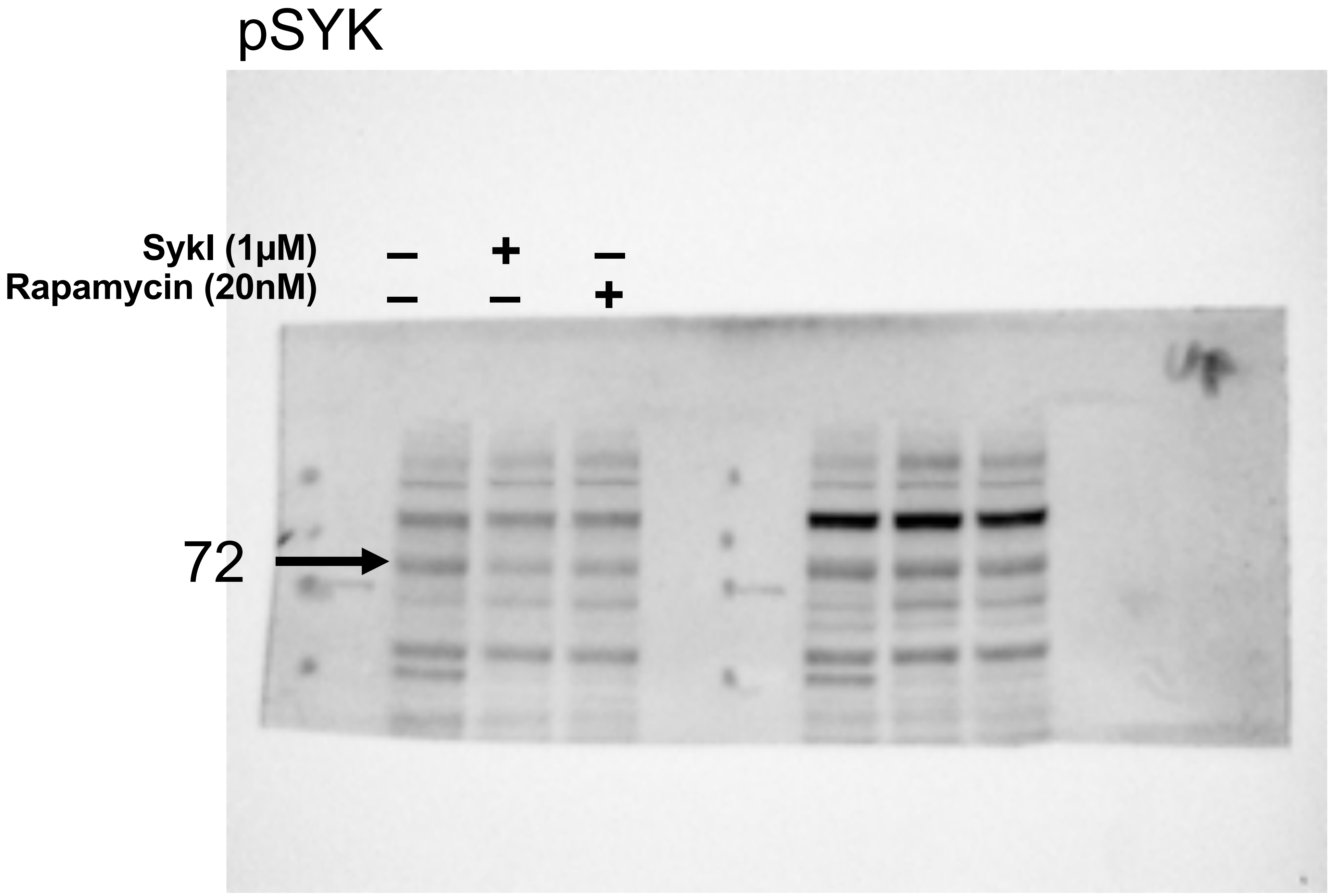

FIGURE 2B

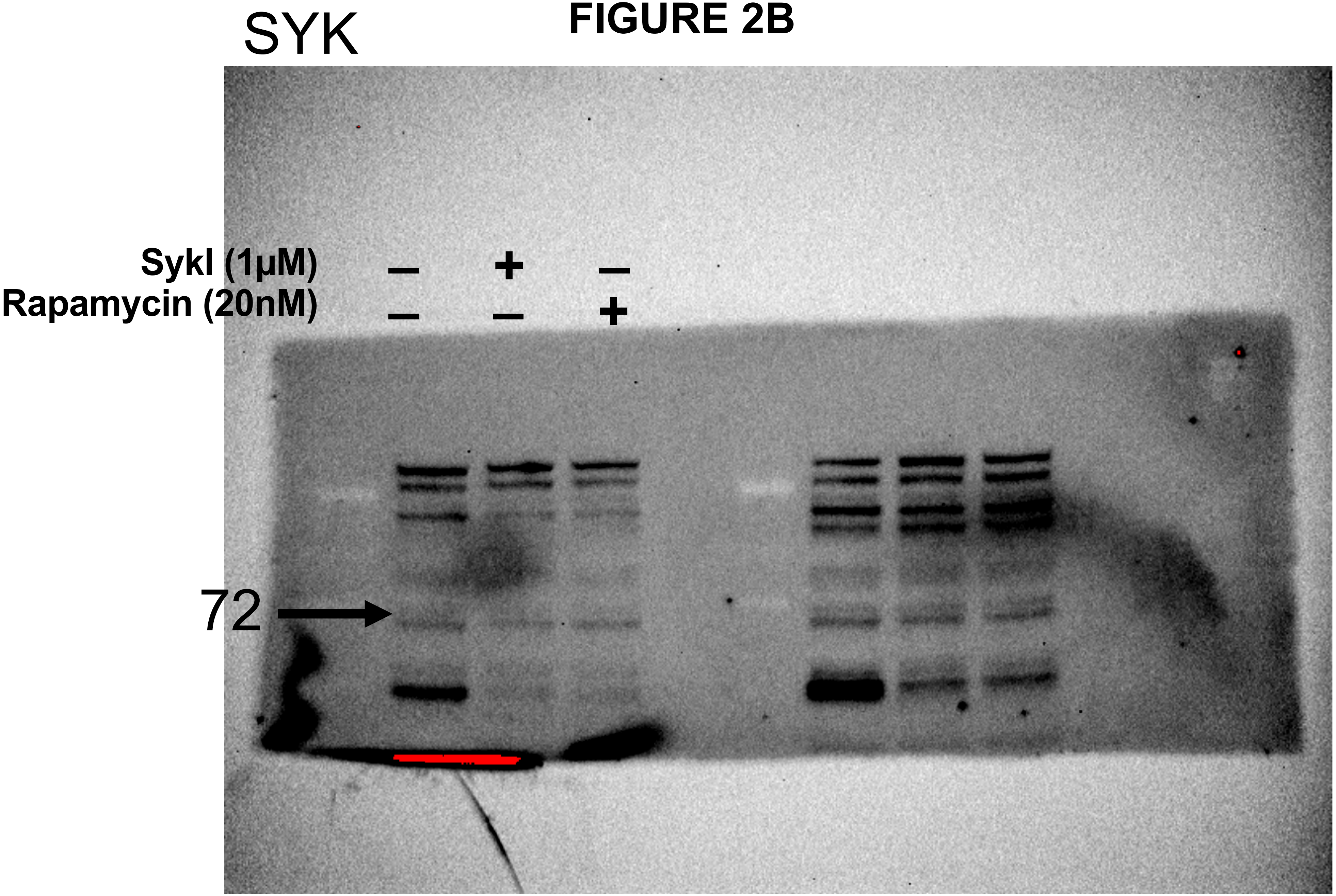

FIGURE 2B

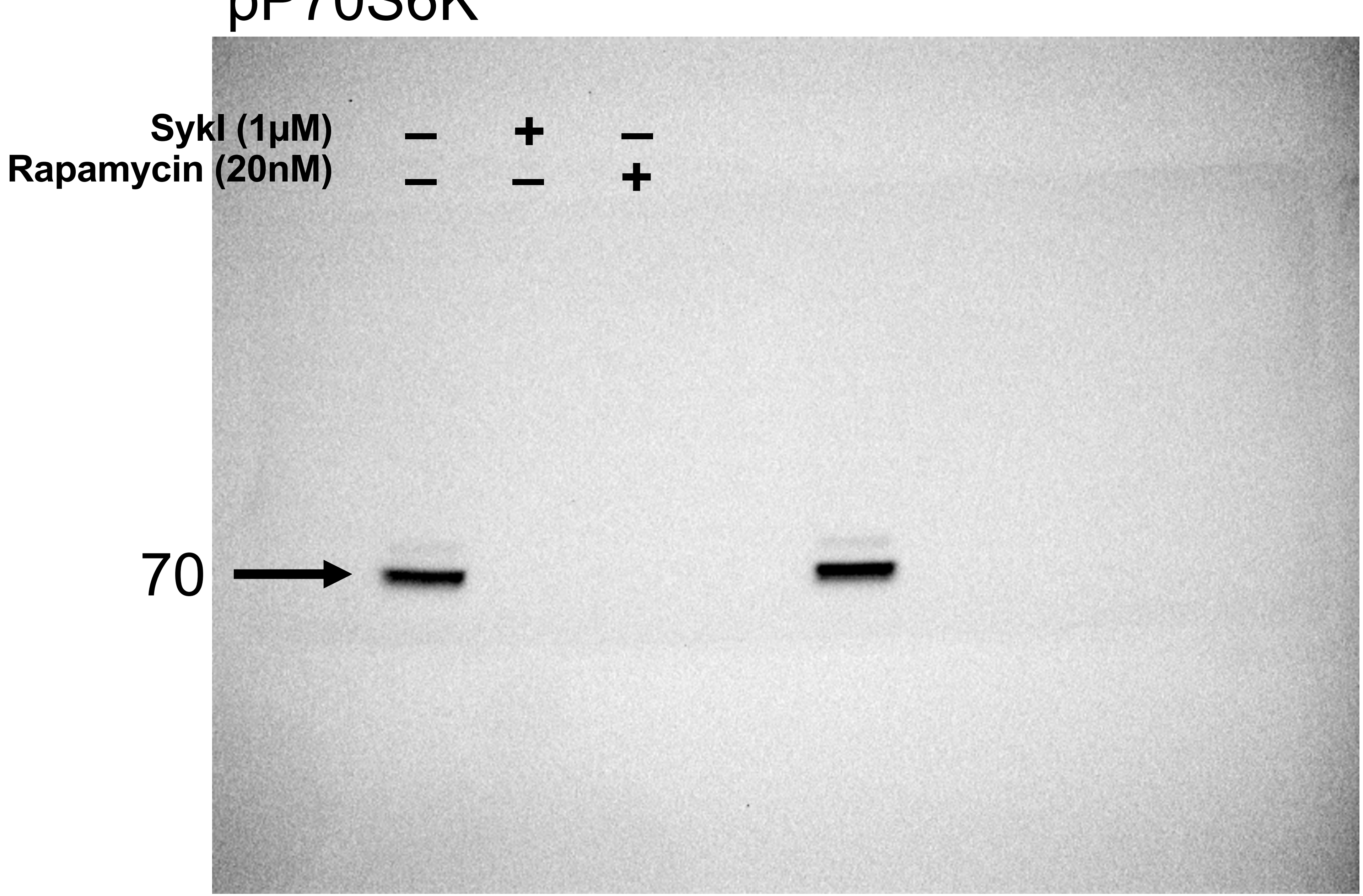

FIGURE 2B

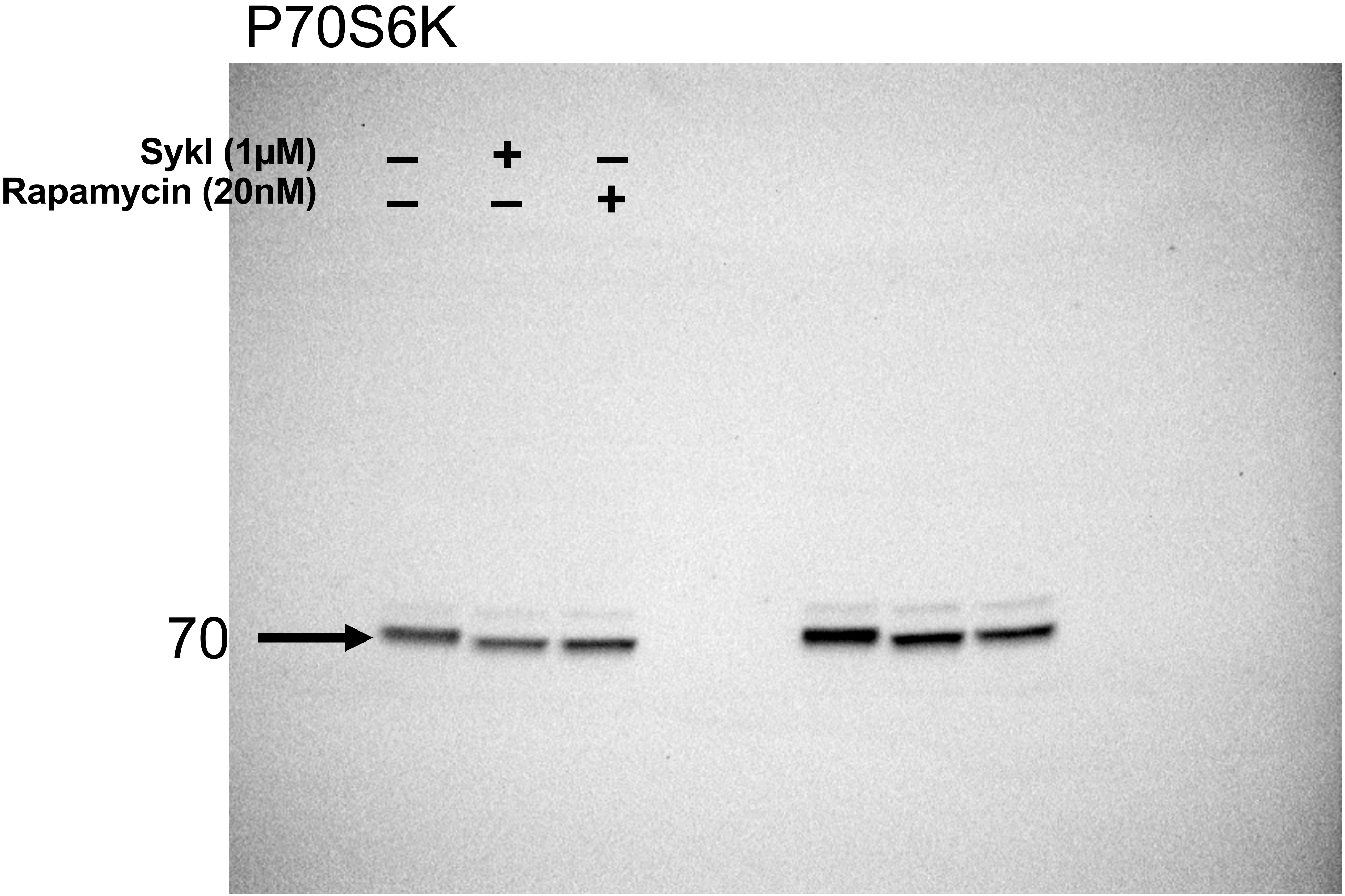

FIGURE 2B

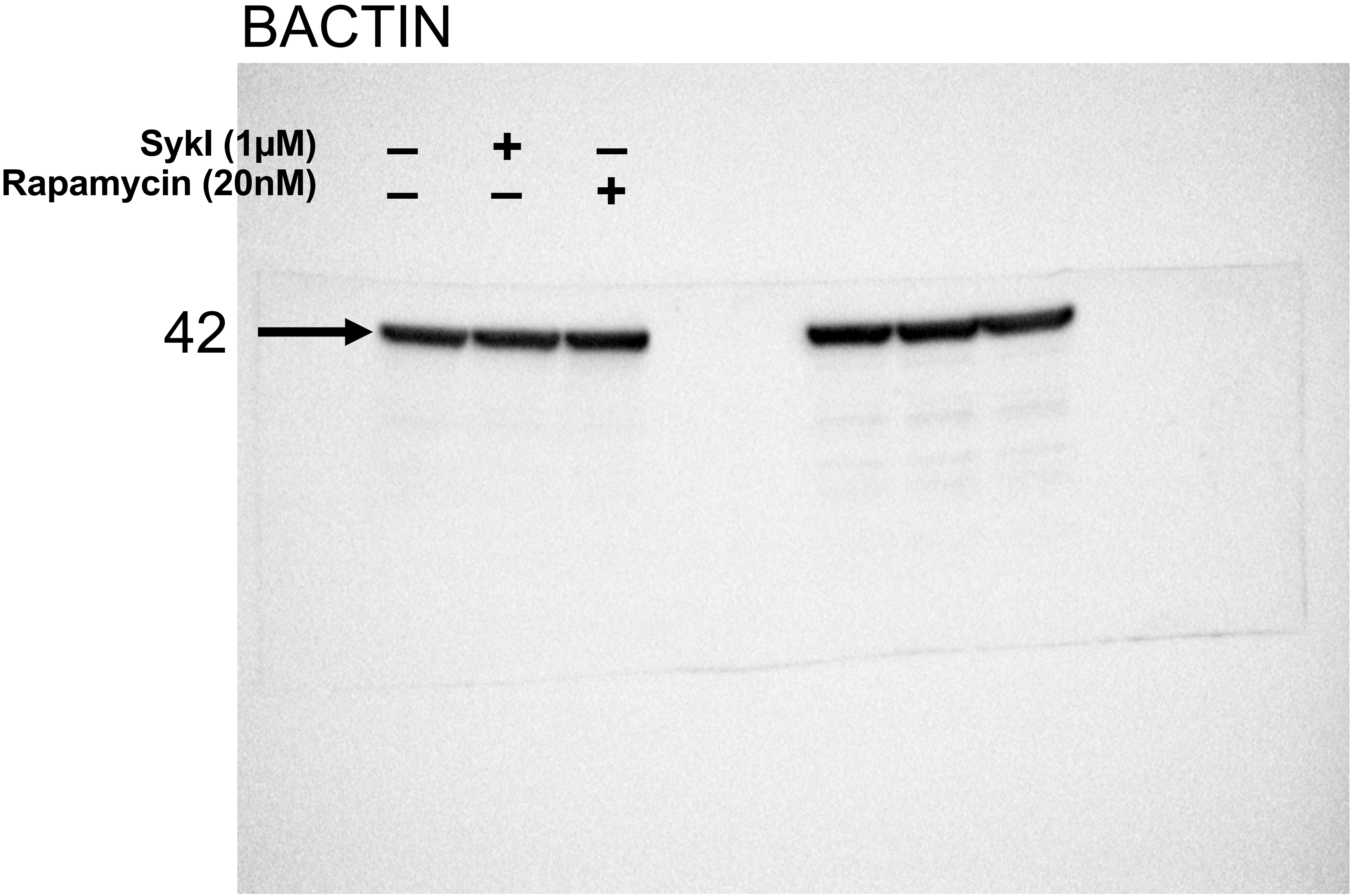

ETV2

FIGURE 2D

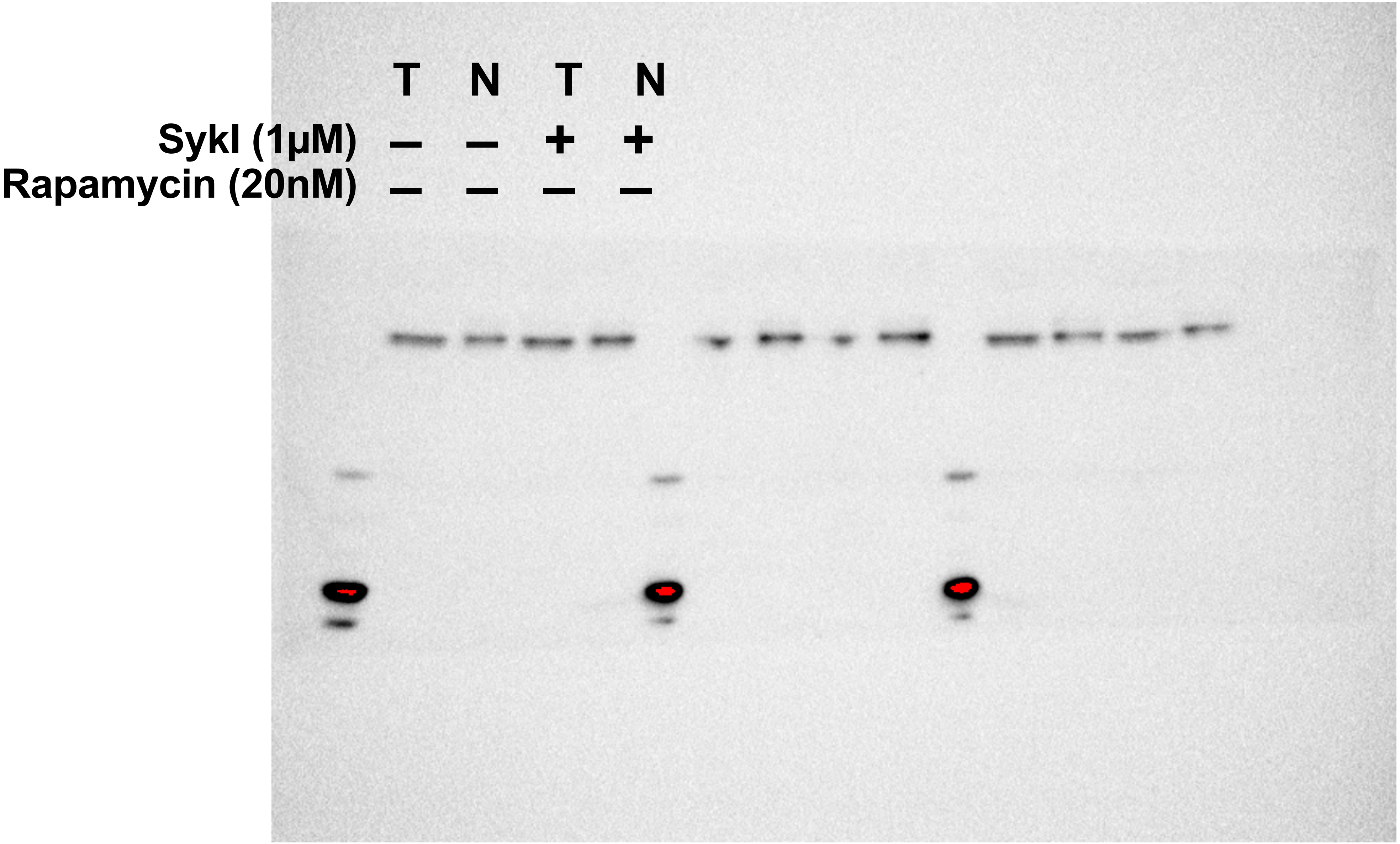

FIGURE 2D

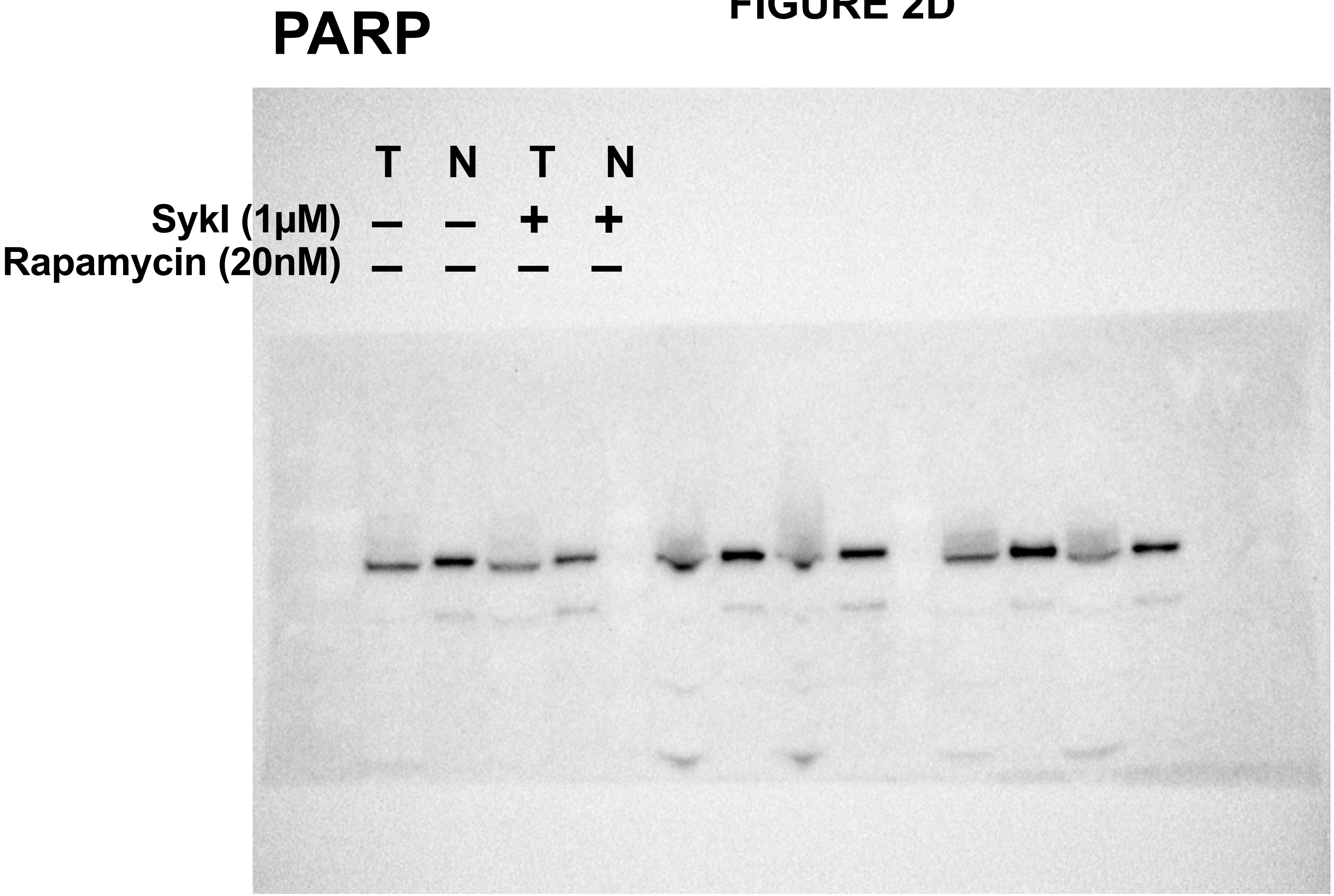

FIGURE 2D

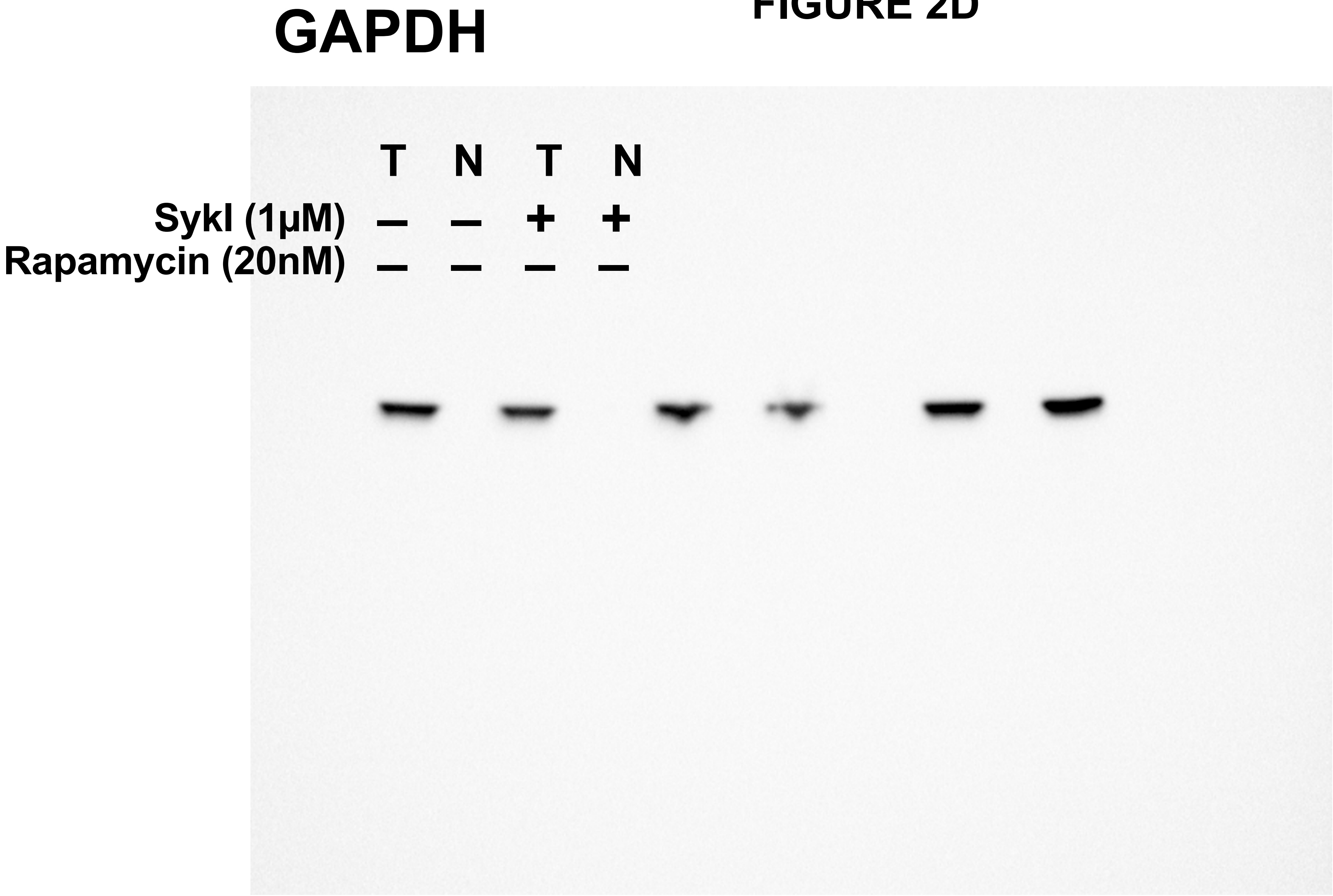

FIGURE 2D

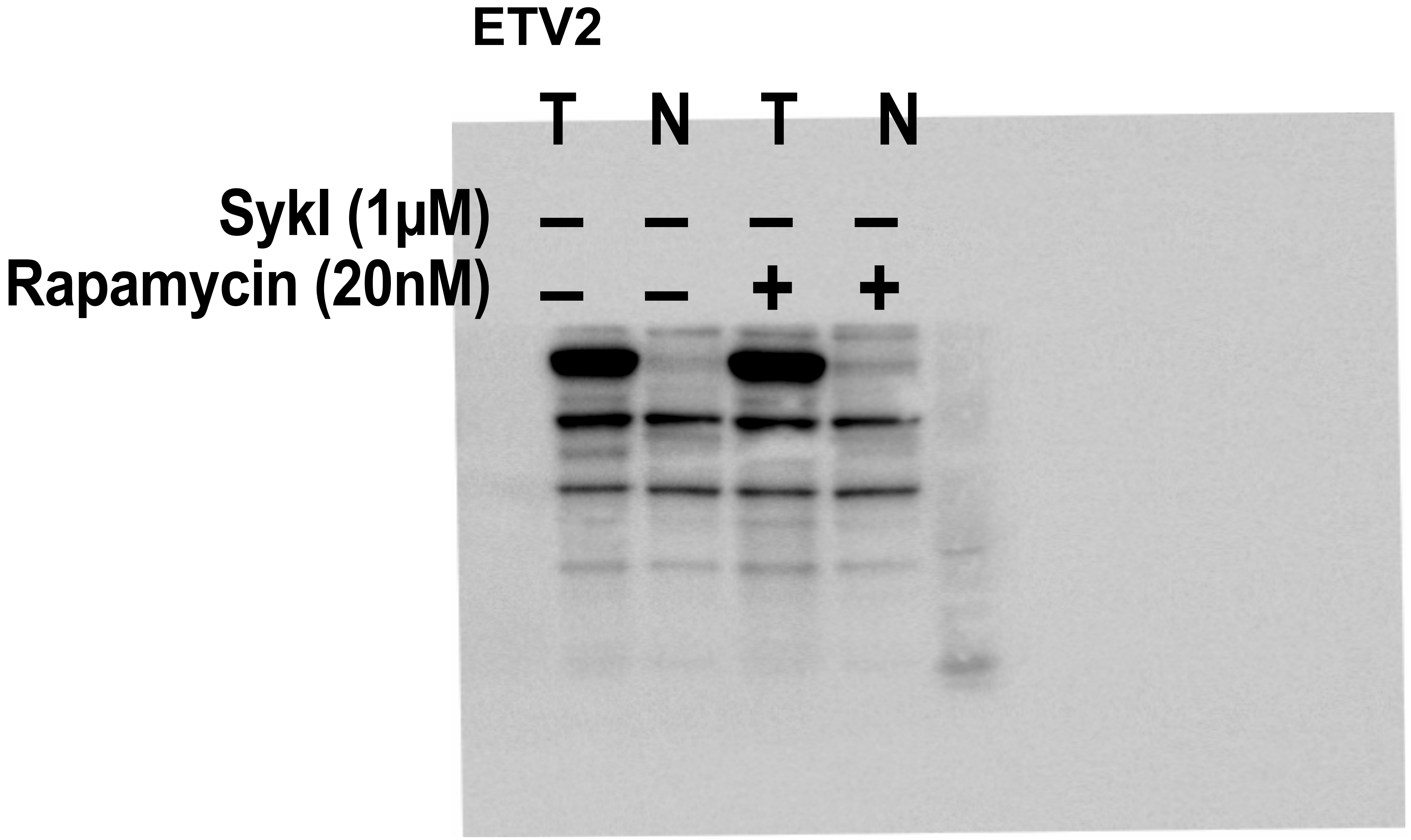

FIGURE 2D

PARP

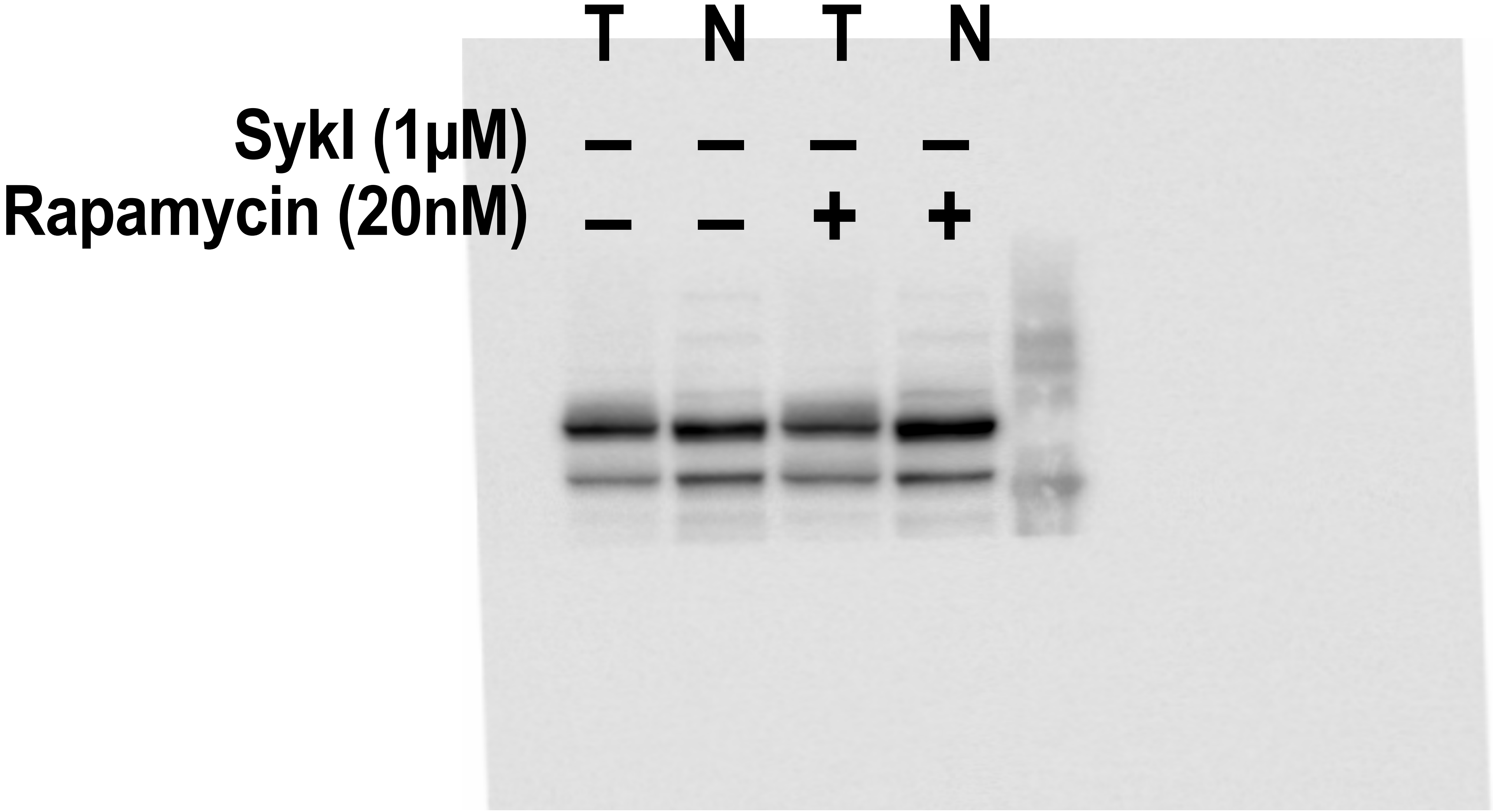

FIGURE 2D

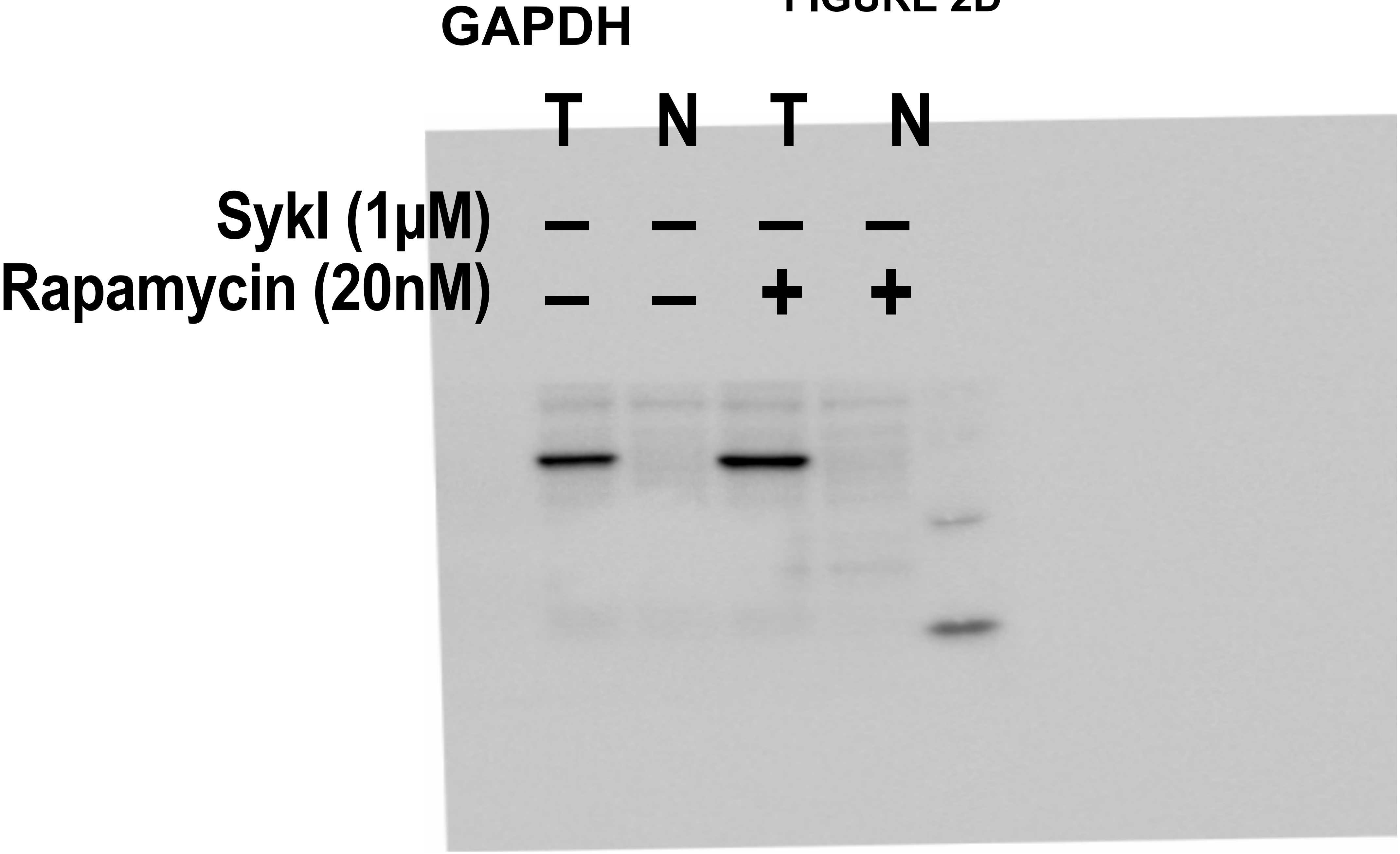

FIGURE 3B

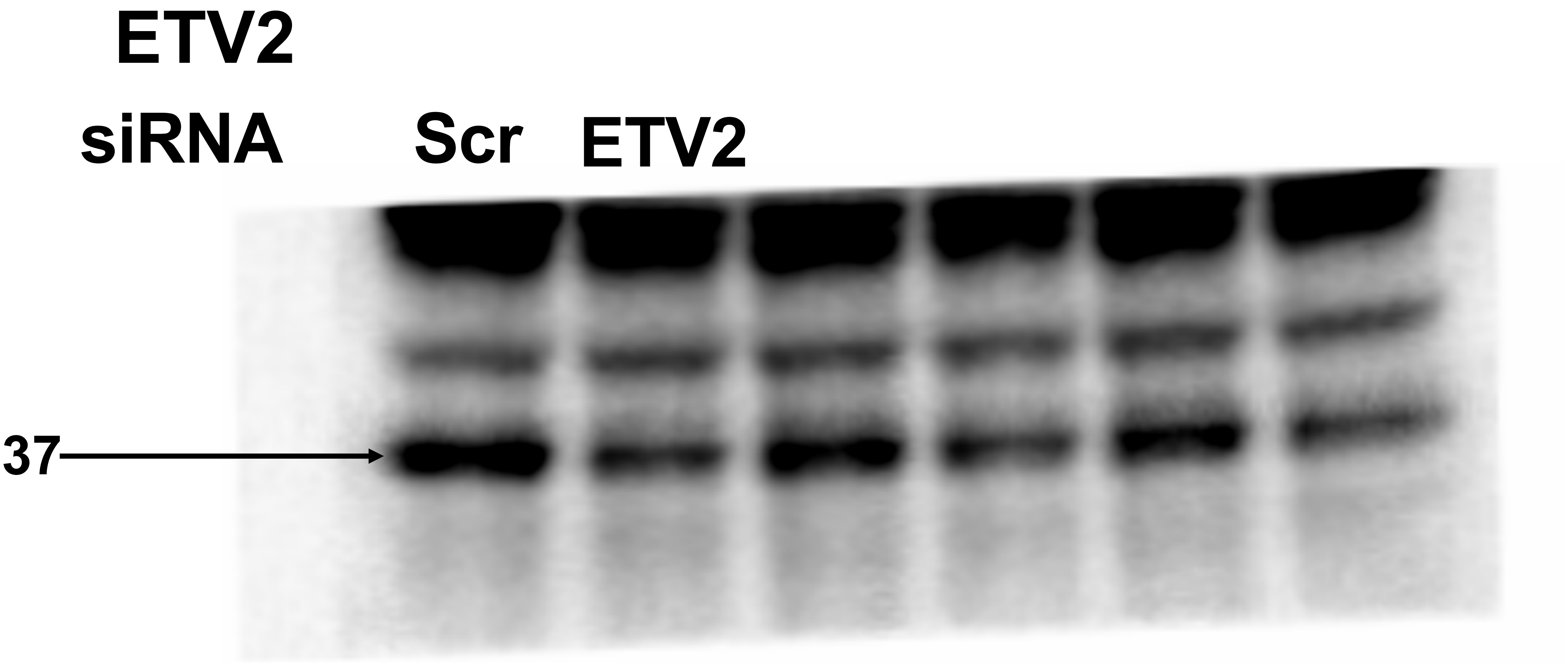

FIGURE 3B

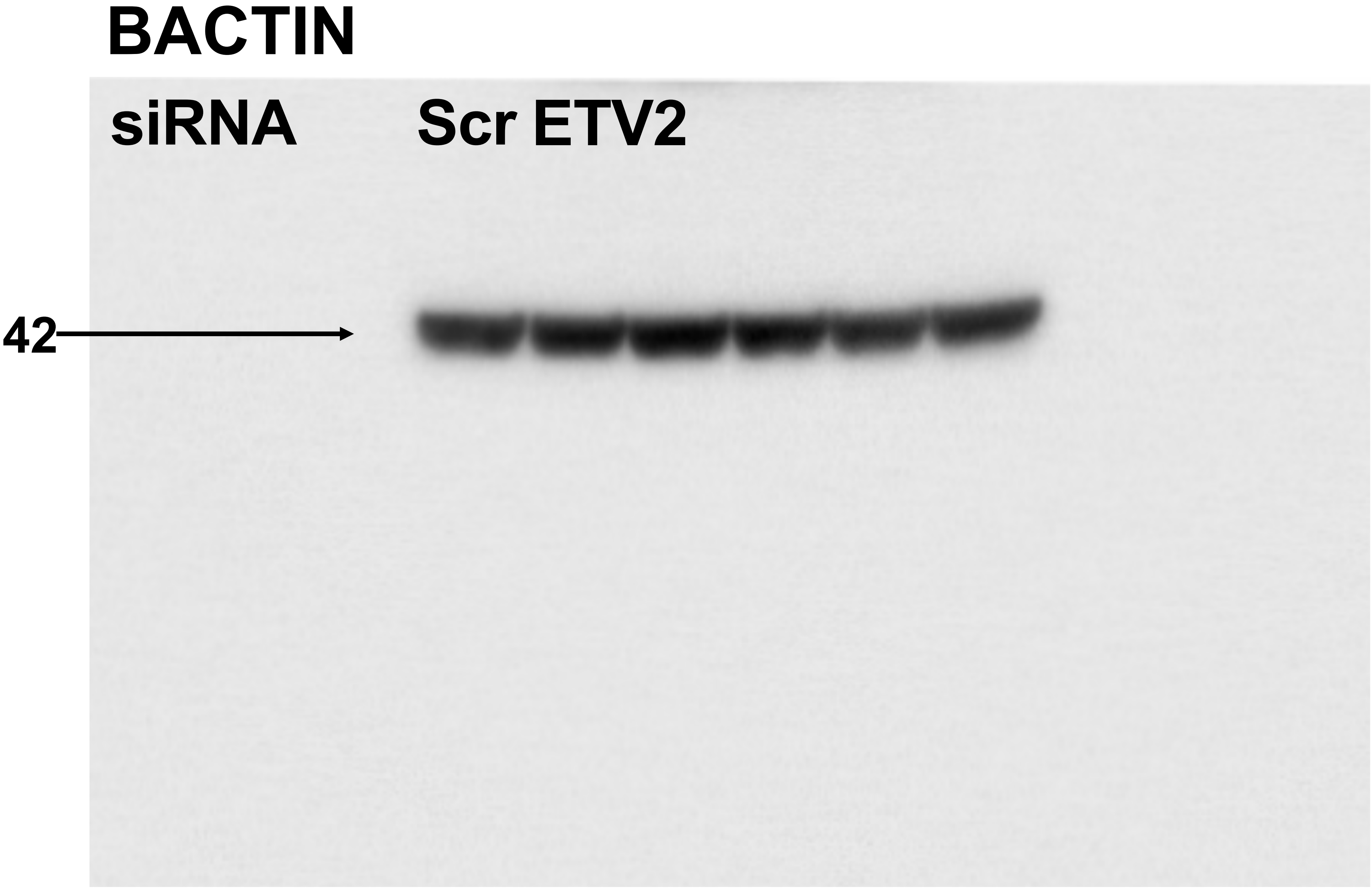

FIGURE 3G

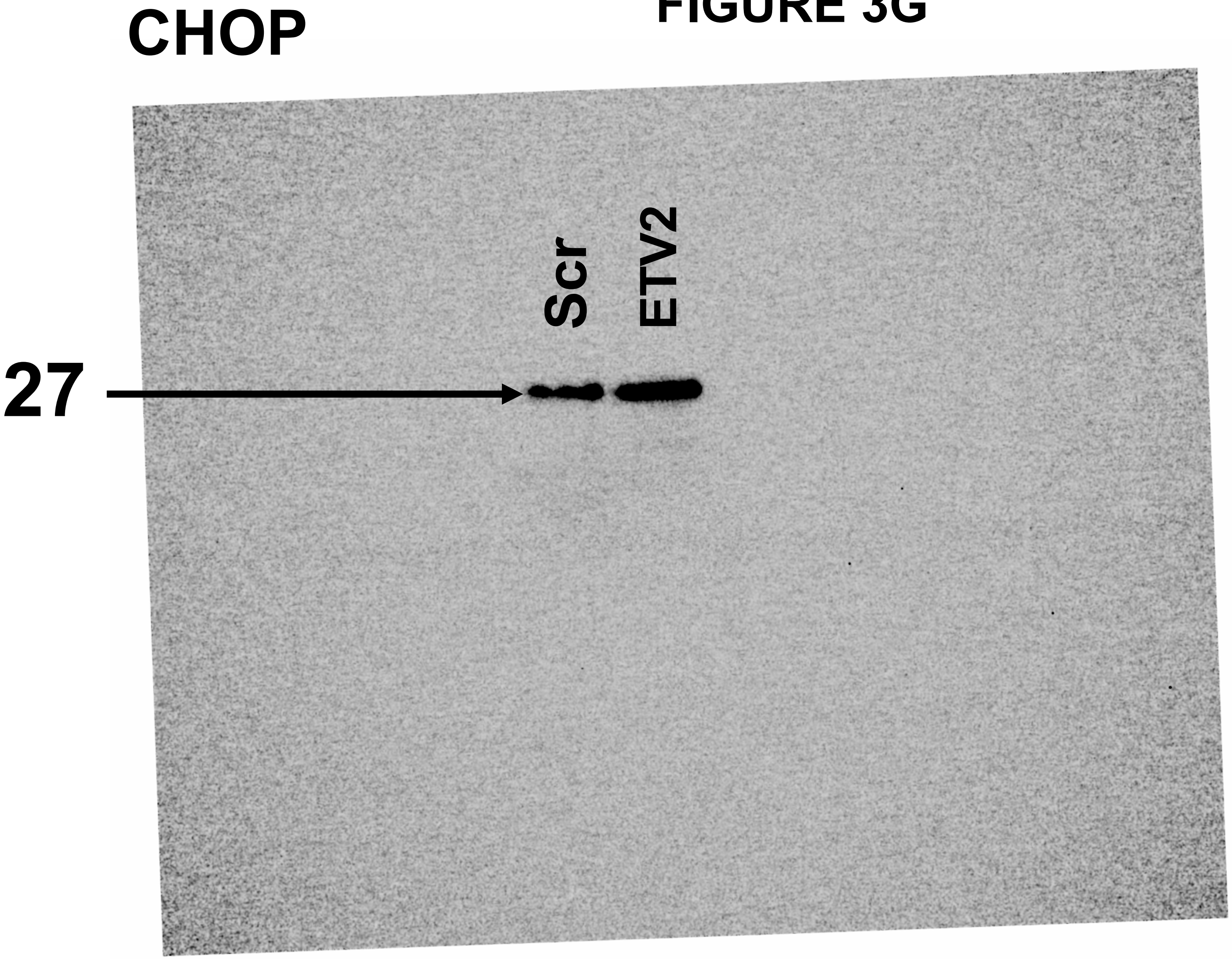

FIGURE 3G

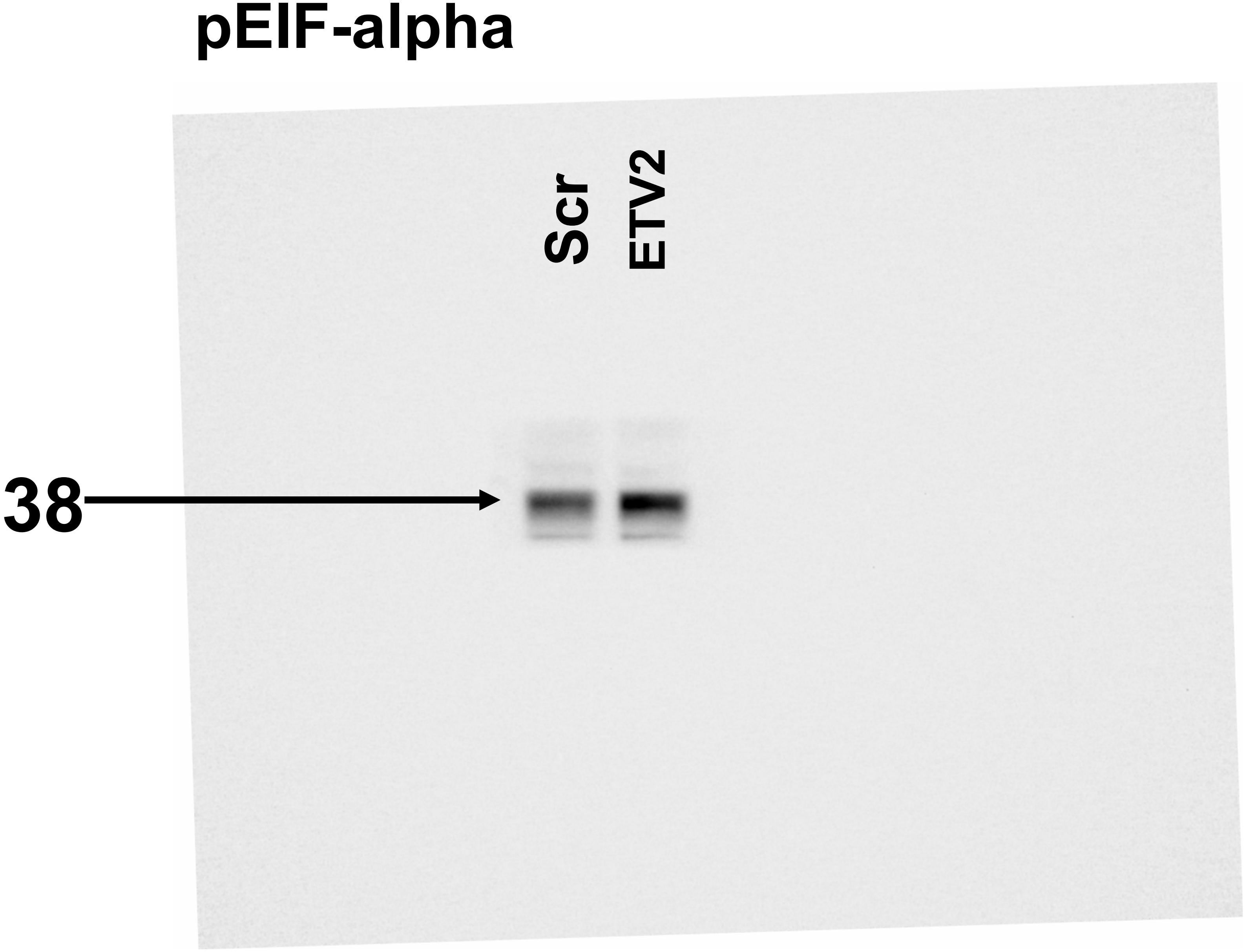

FIGURE 3G

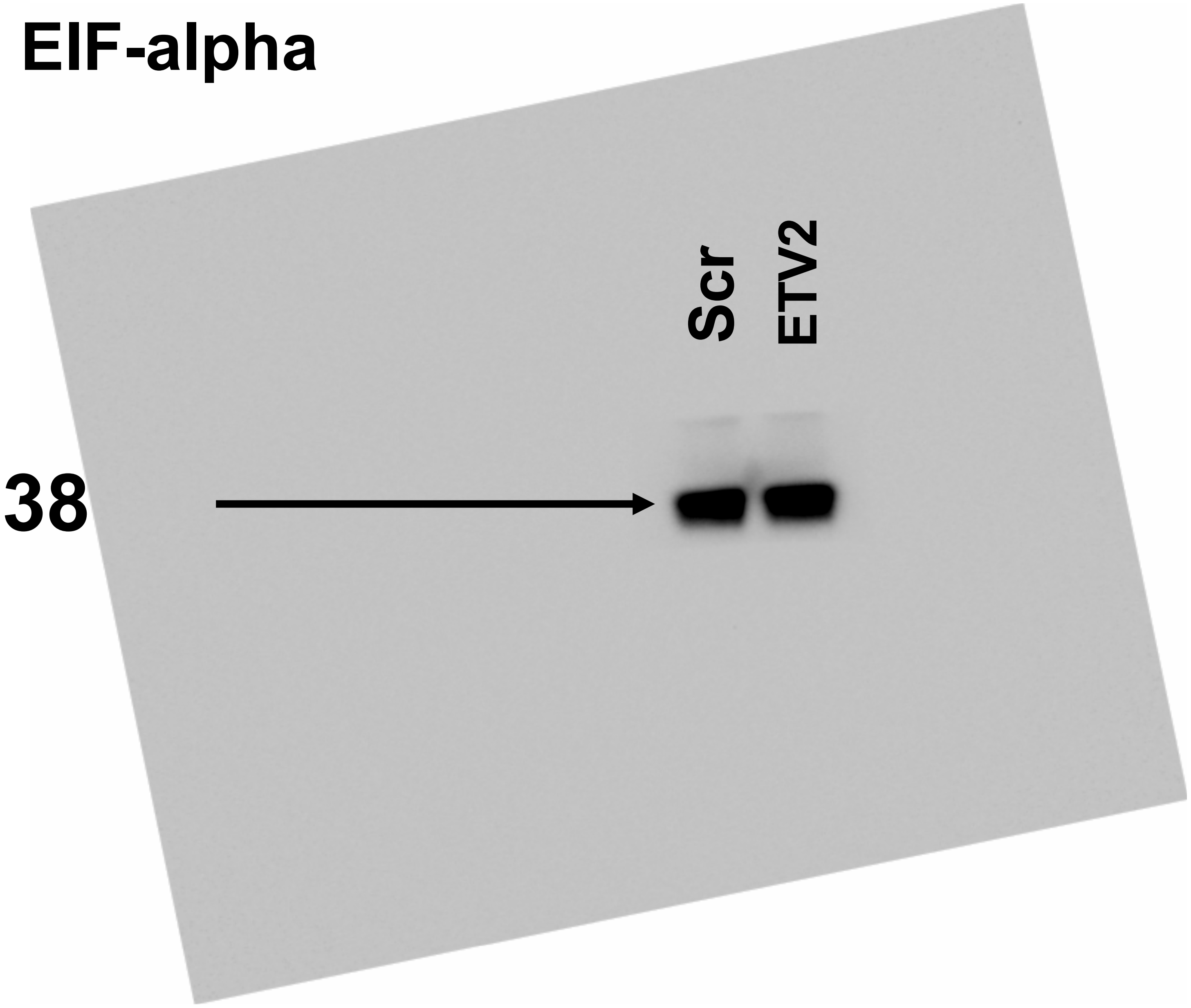

FIGURE 3G

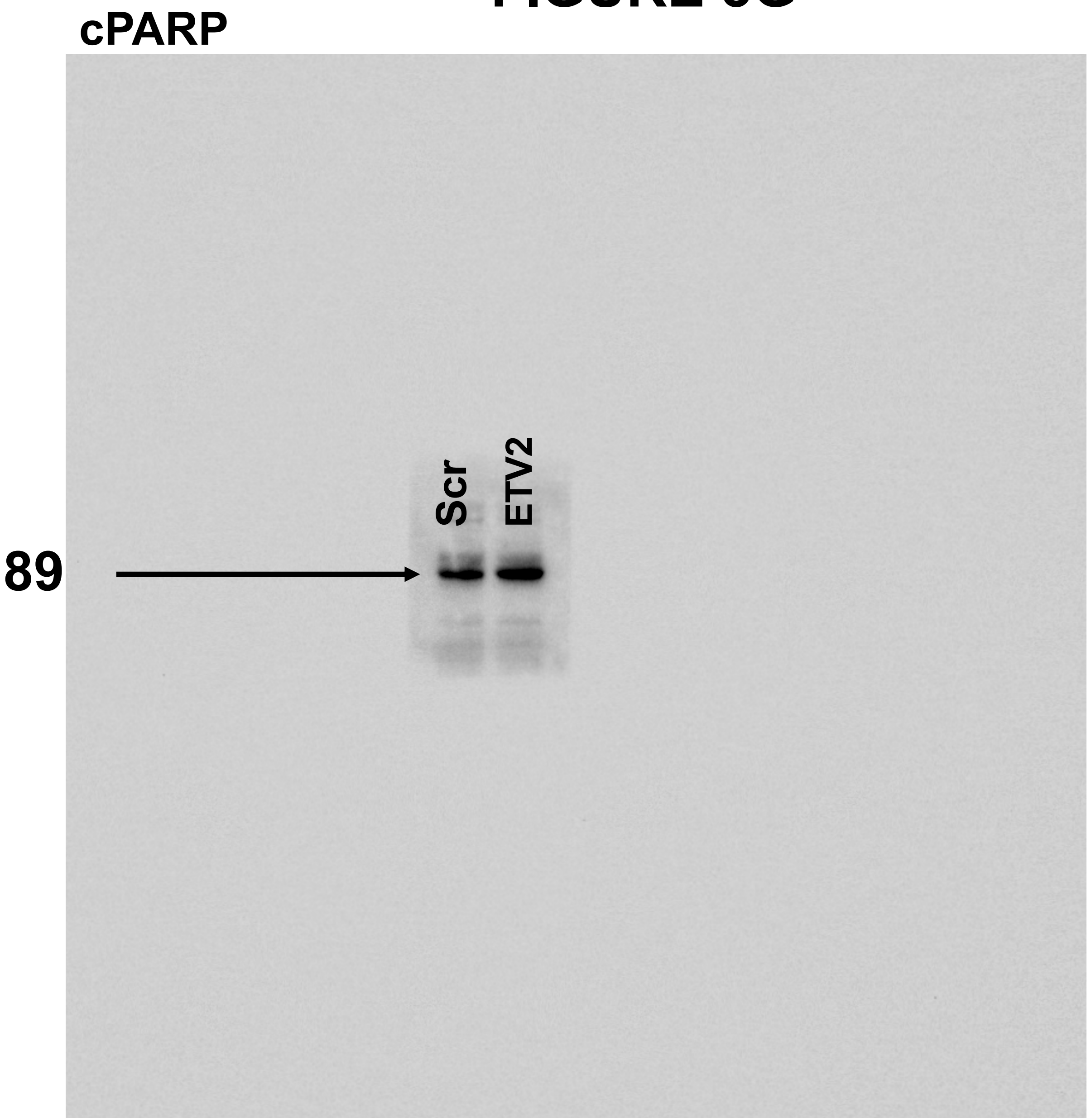

FIGURE 3G

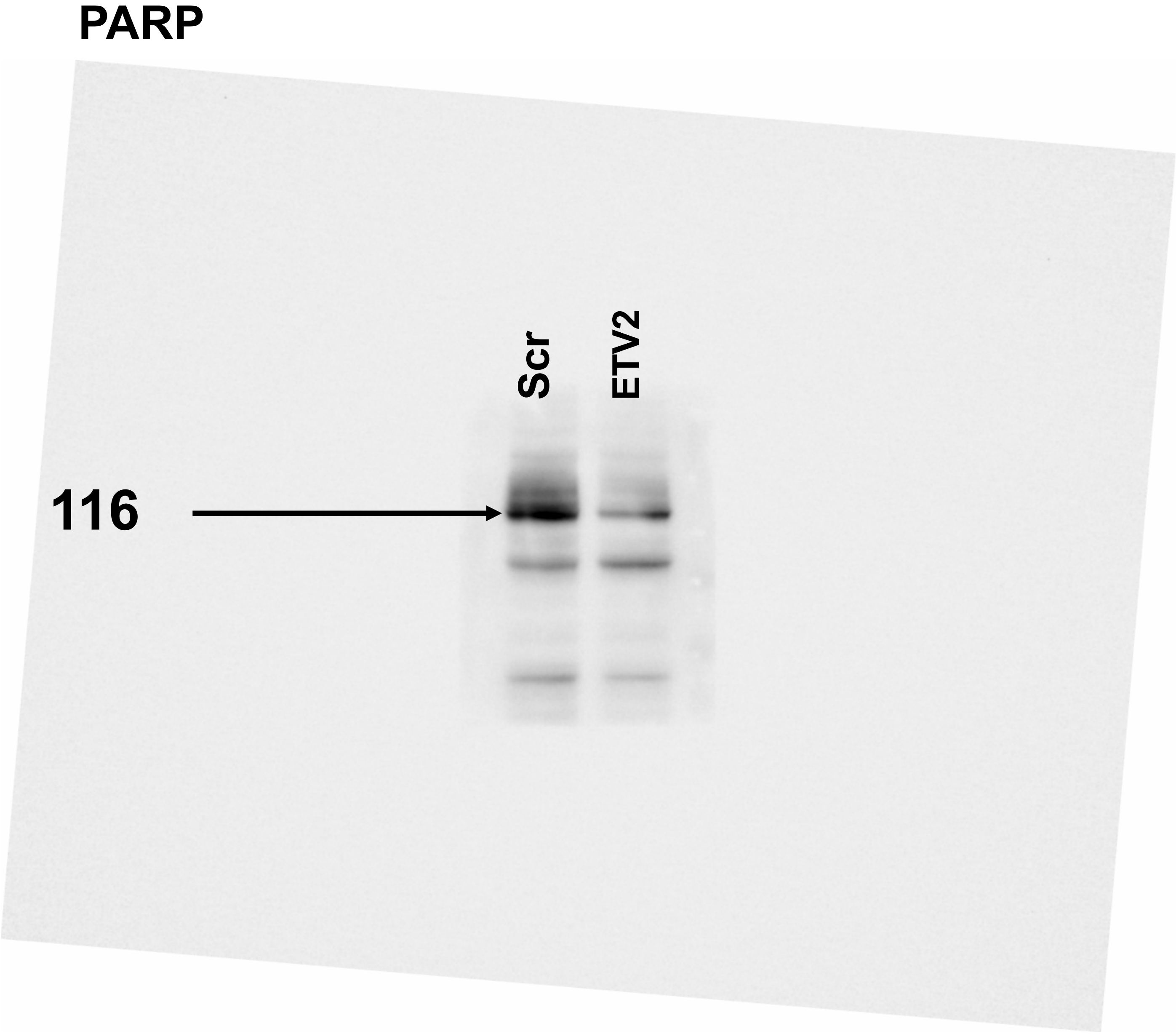

FIGURE 3G

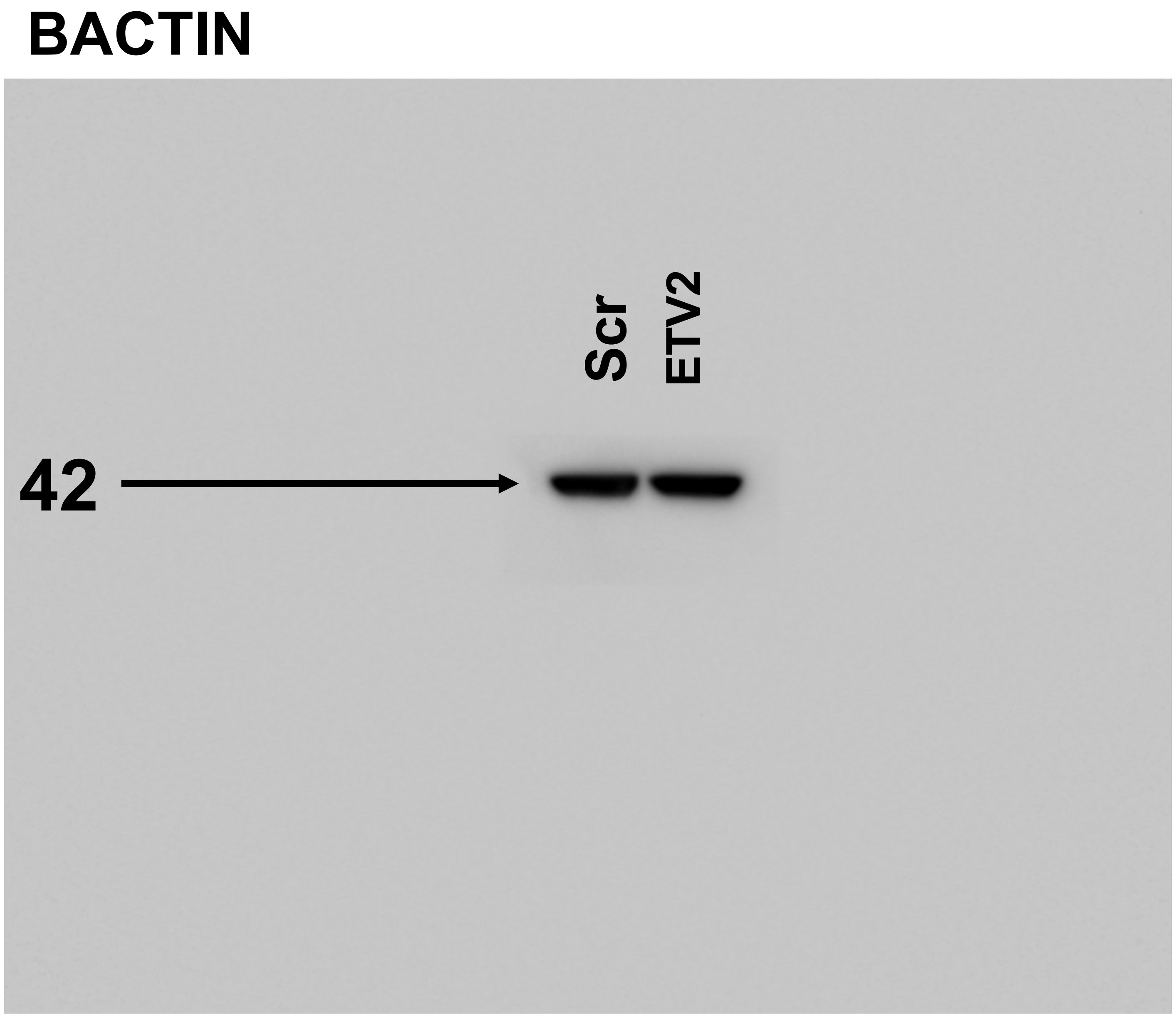

FIGURE 4D

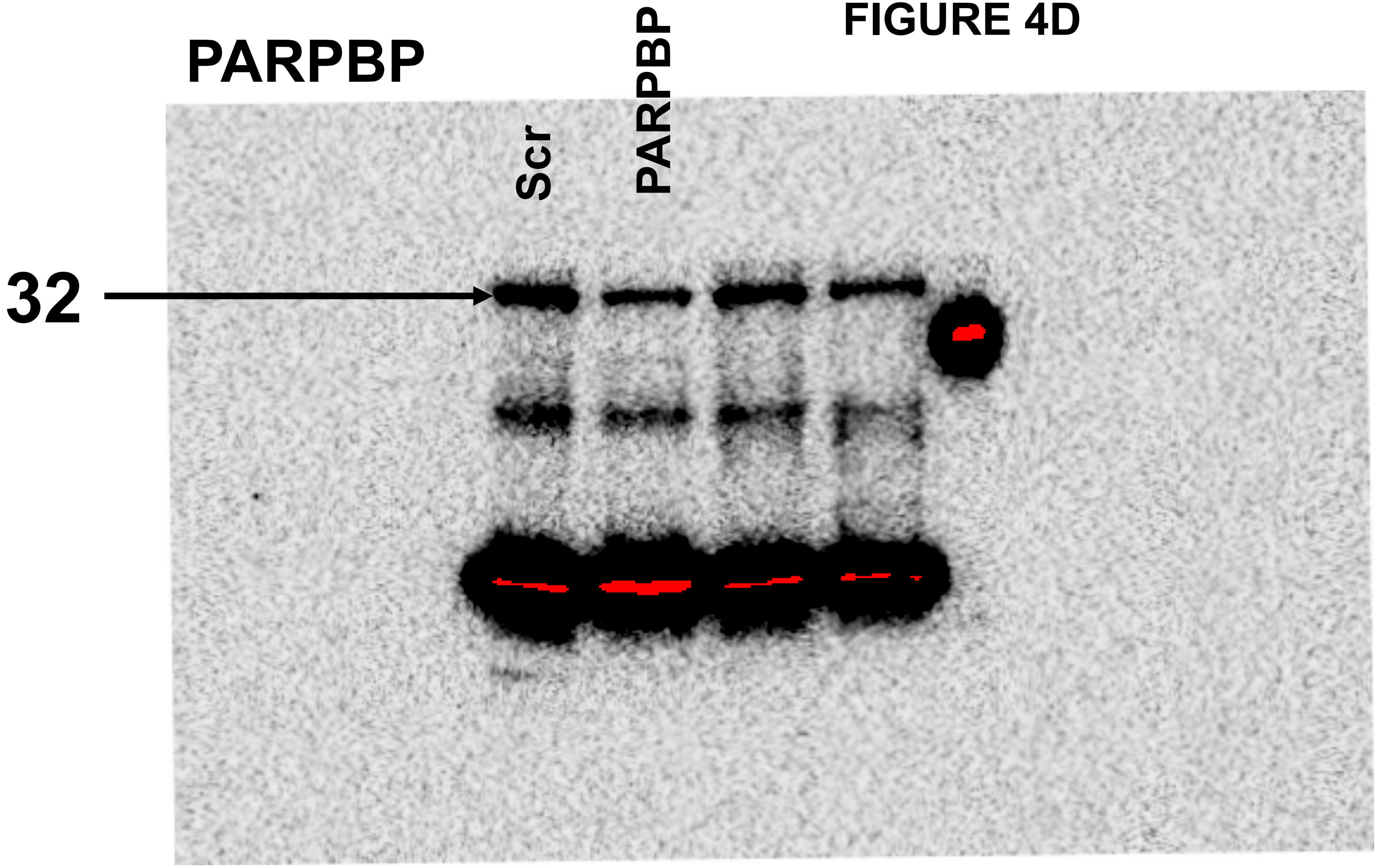

FIGURE 4D

BACTIN

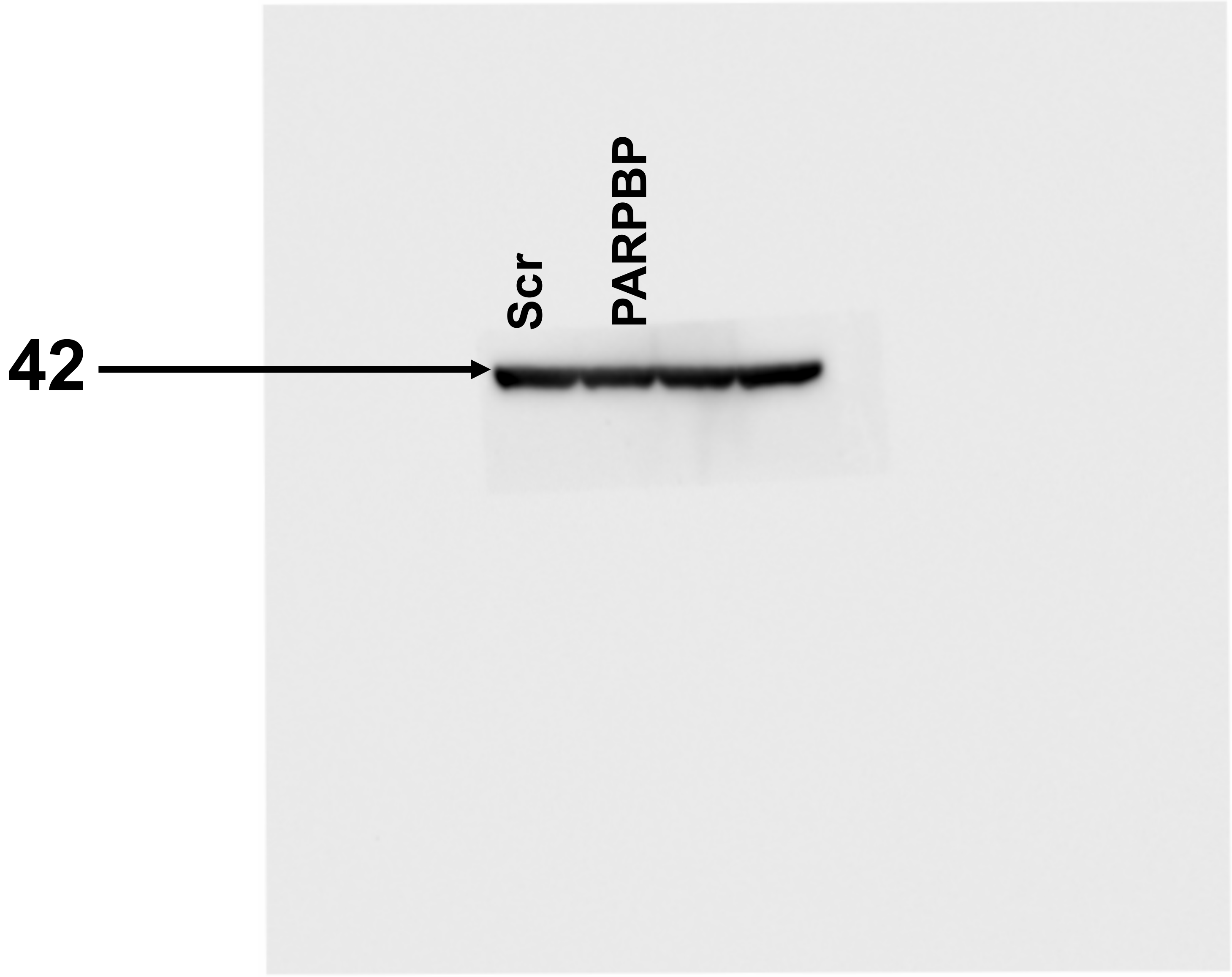

FIGURE 4F

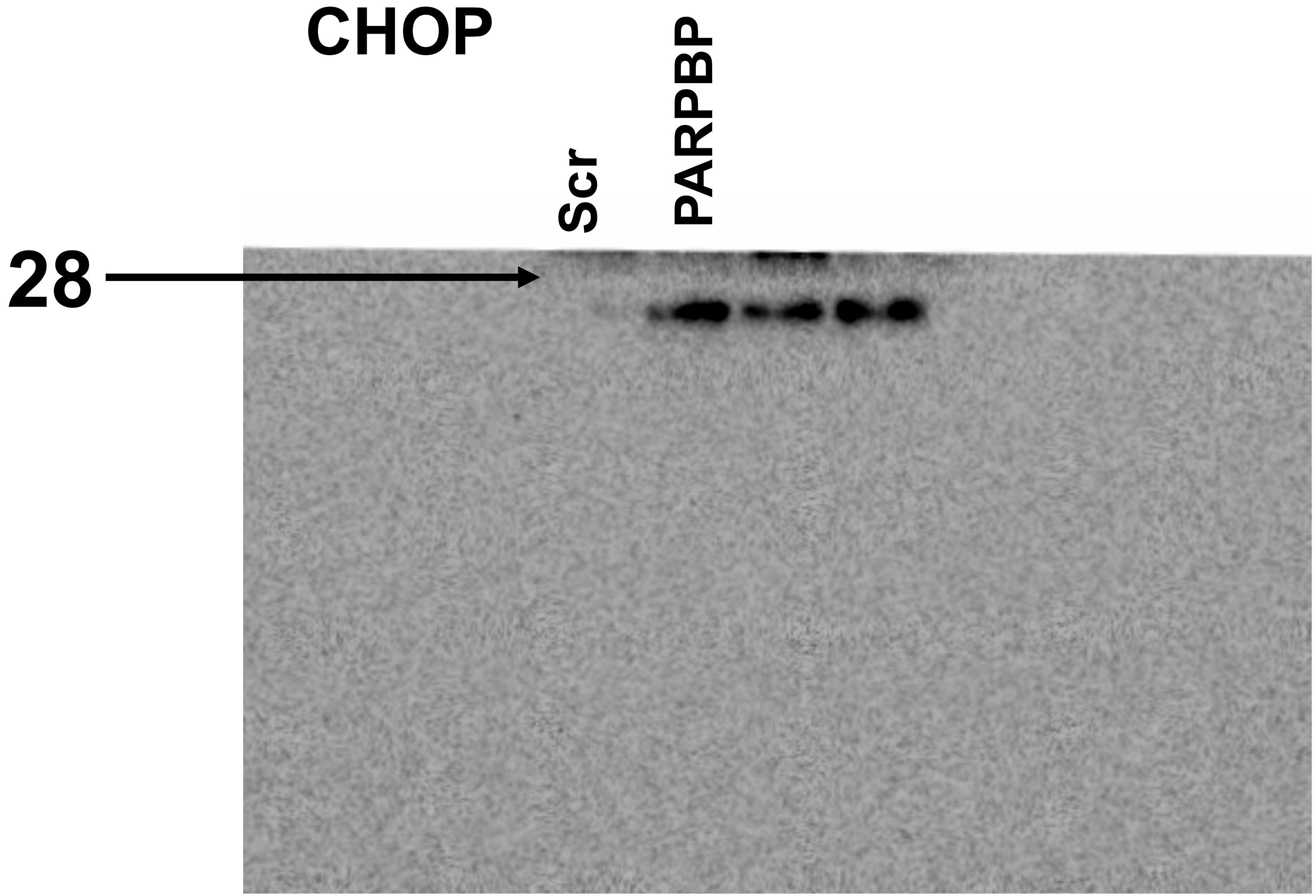

FIGURE 4F

pEIF-alpha

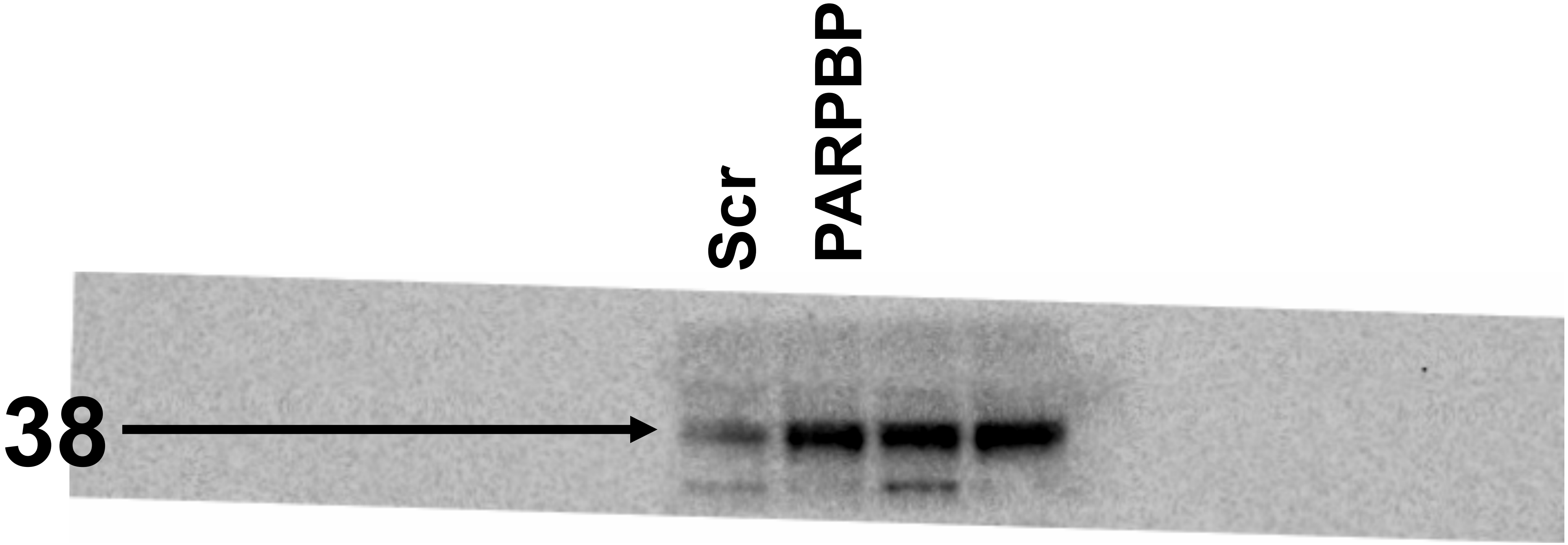

FIGURE 4F

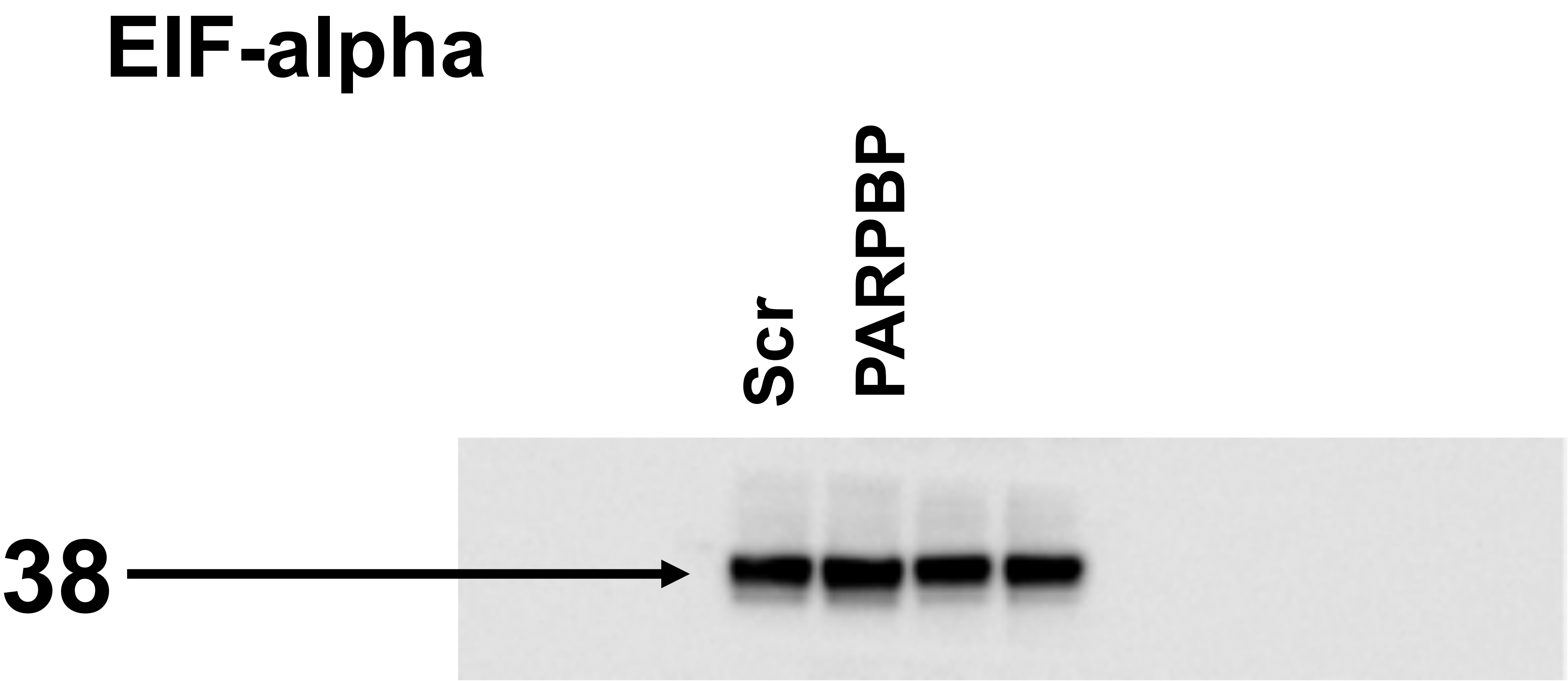

FIGURE 4F

PARP (116) and cPARP (89)

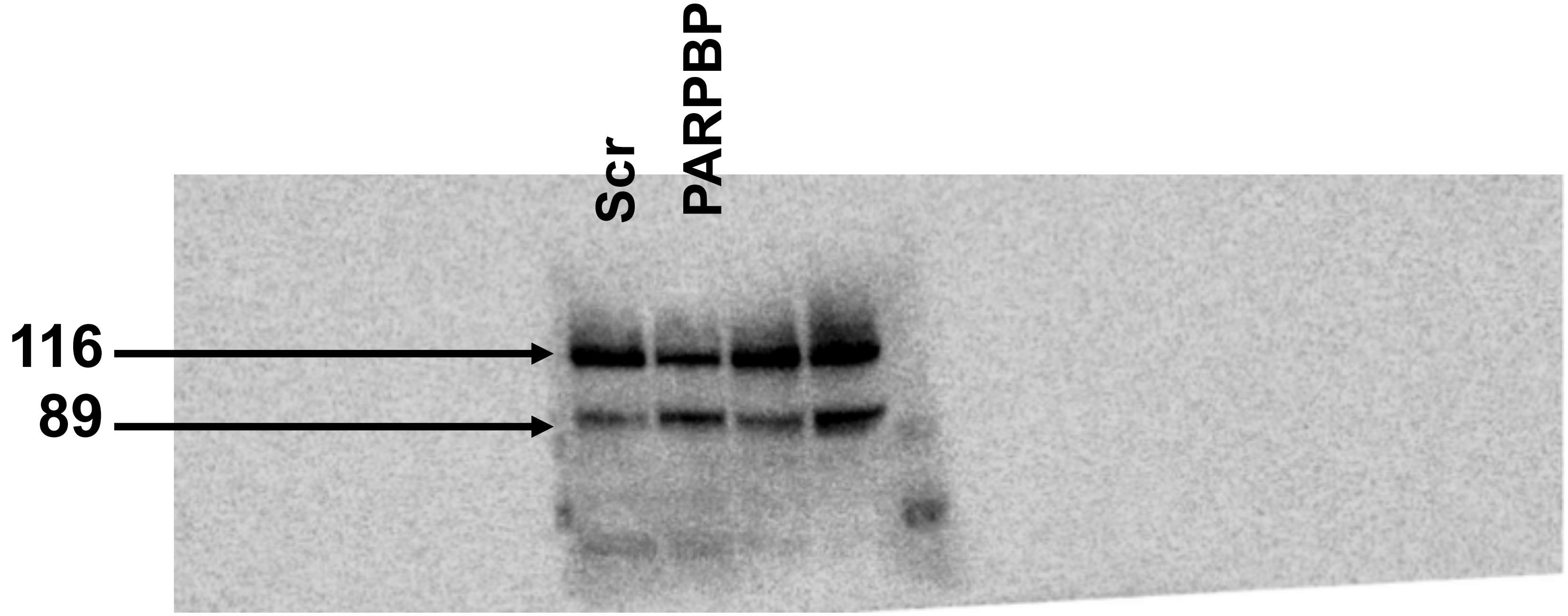

FIGURE 4F

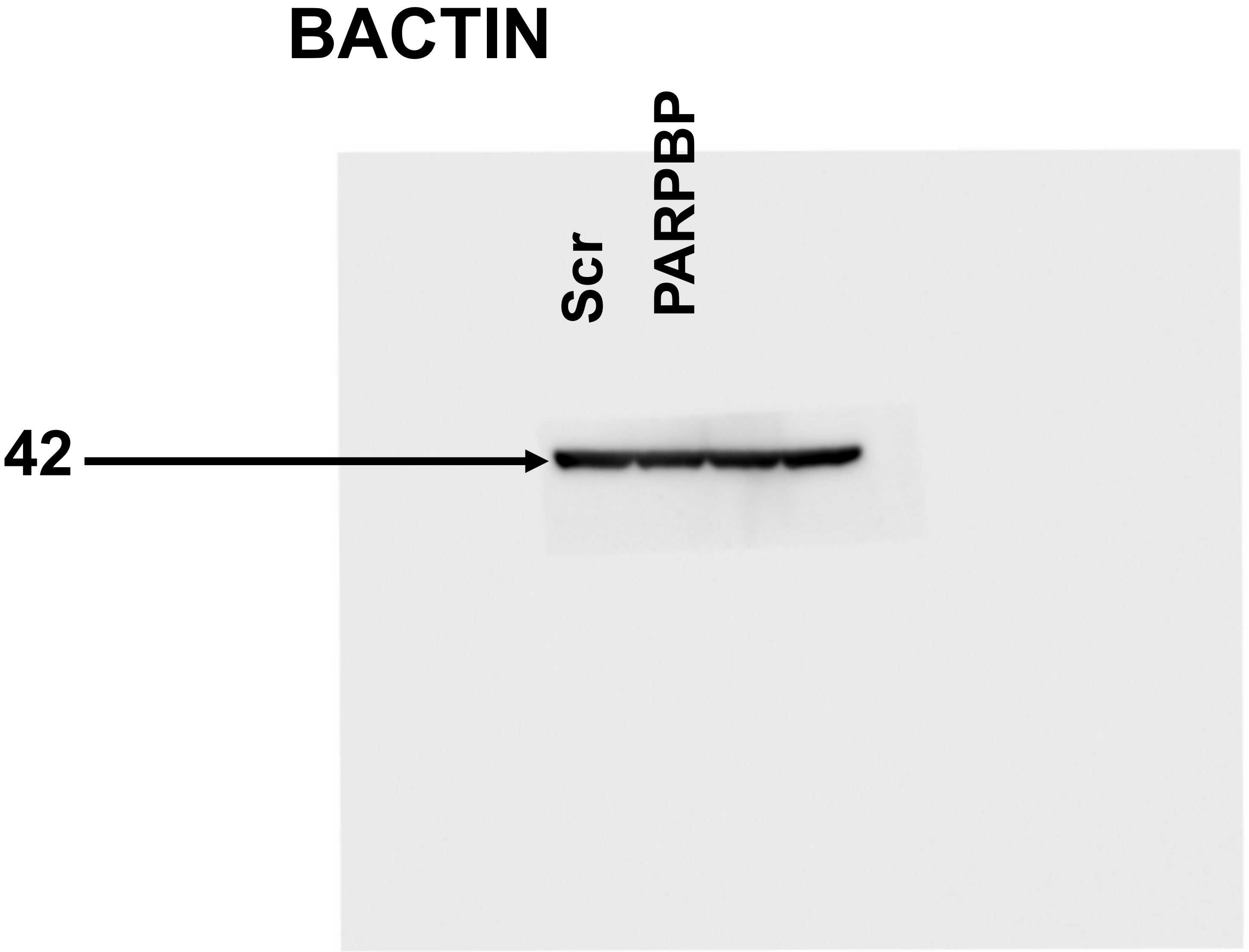

FIGURE 6

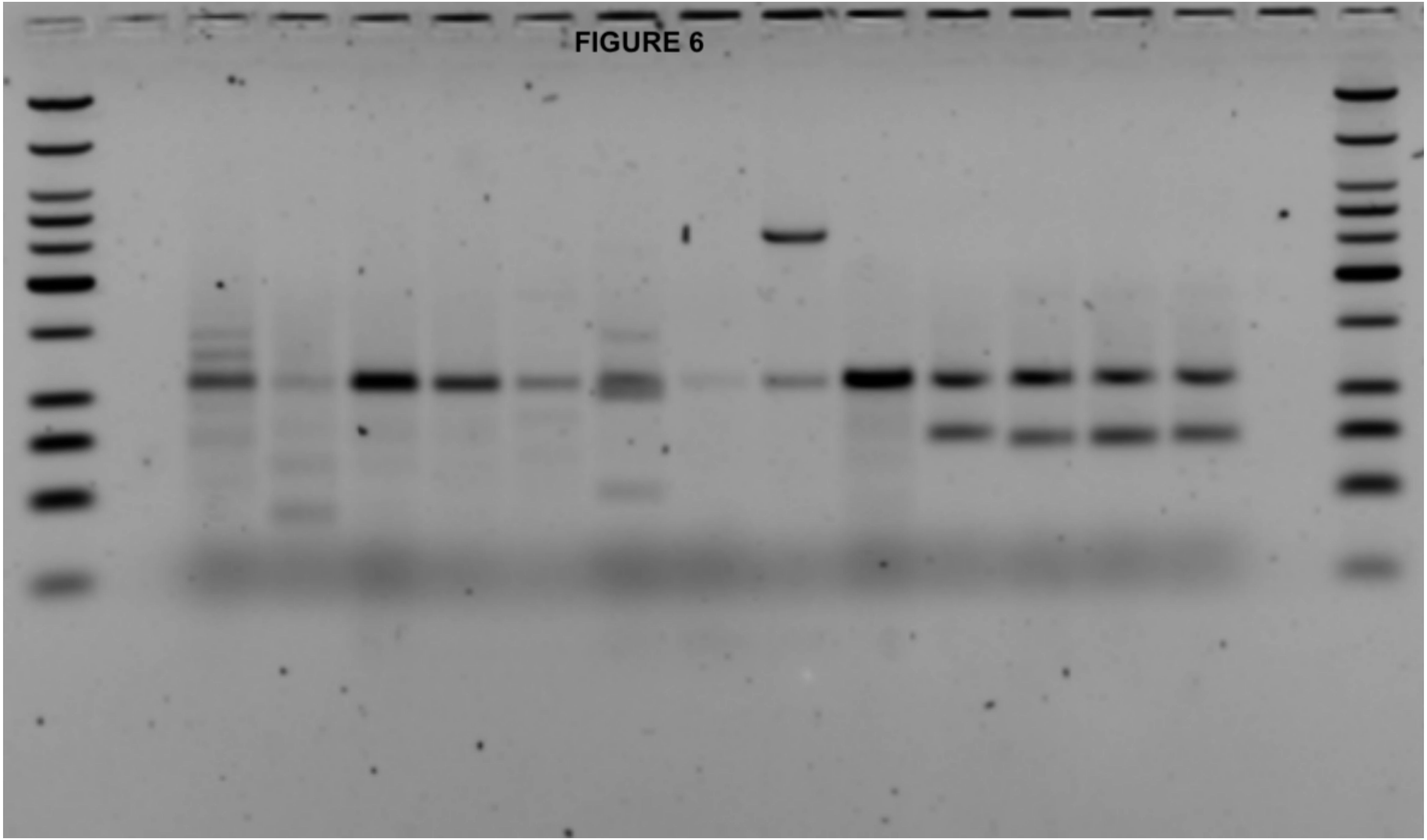

SUPP FIGURE 2B

BACTIN

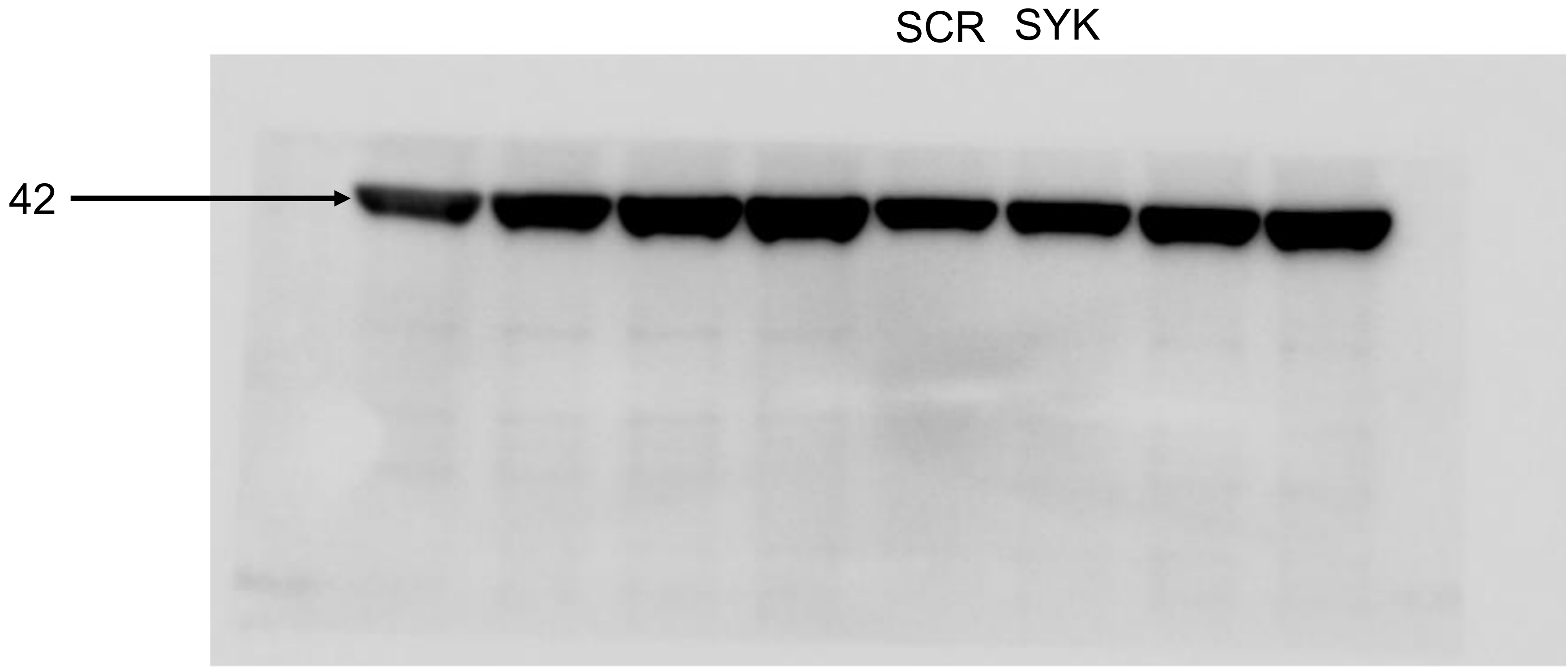

SUPP FIGURE 2B

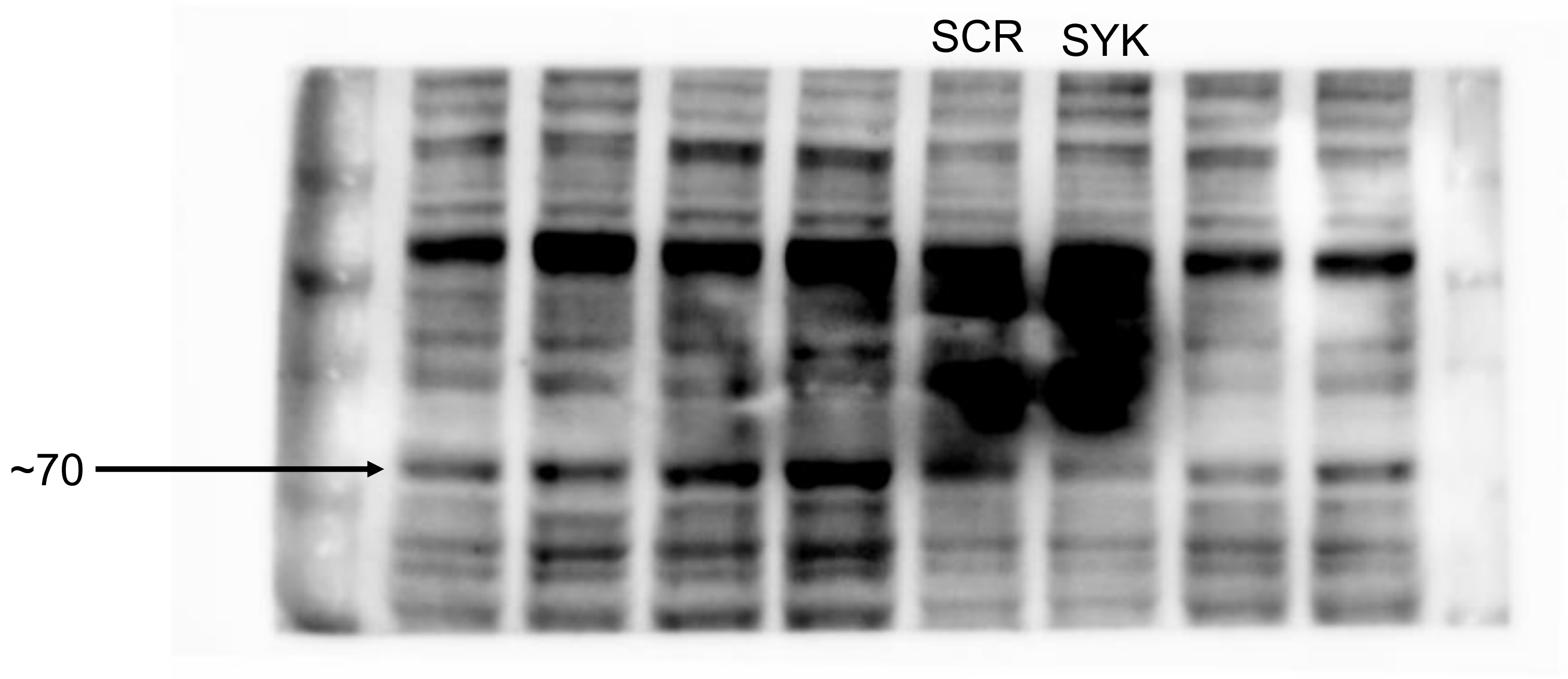

SUPP FIGURE 2D

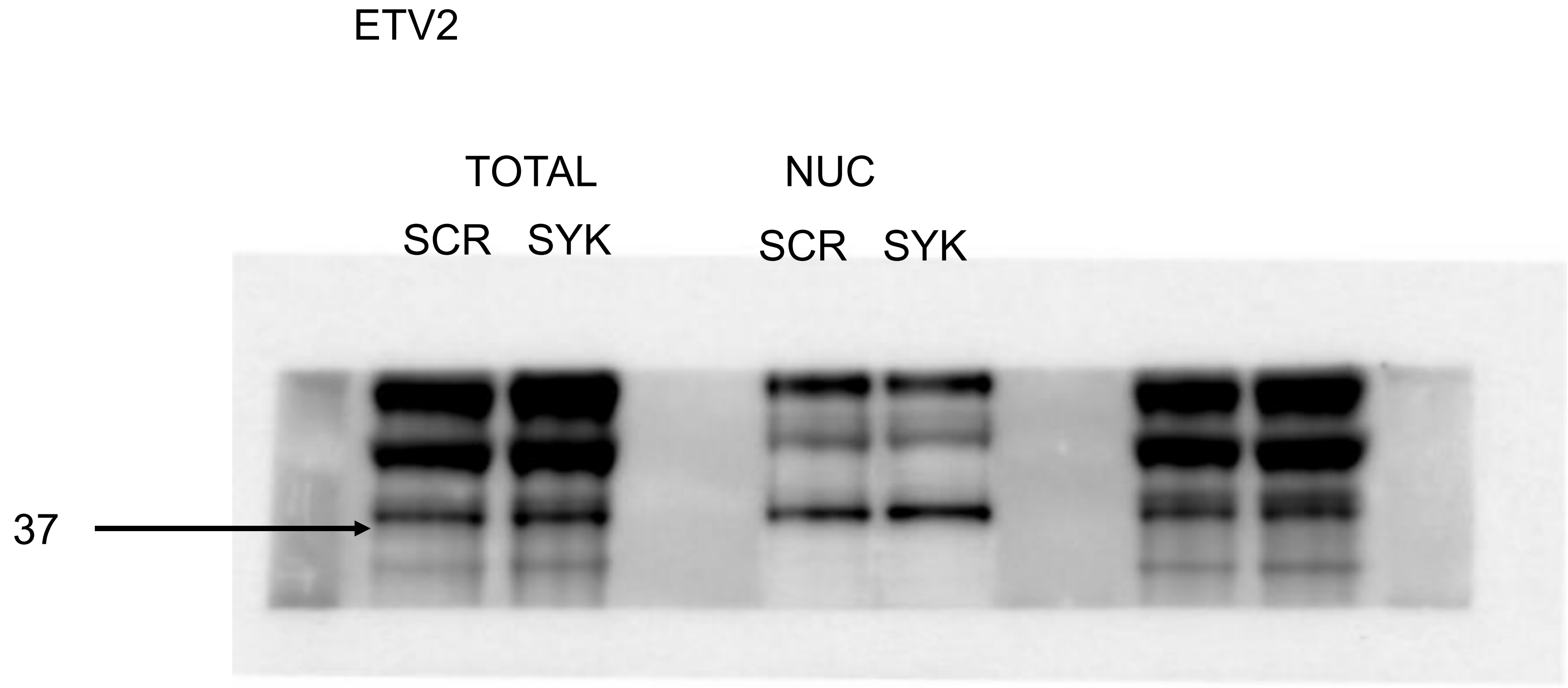

SUPP FIGURE 2D

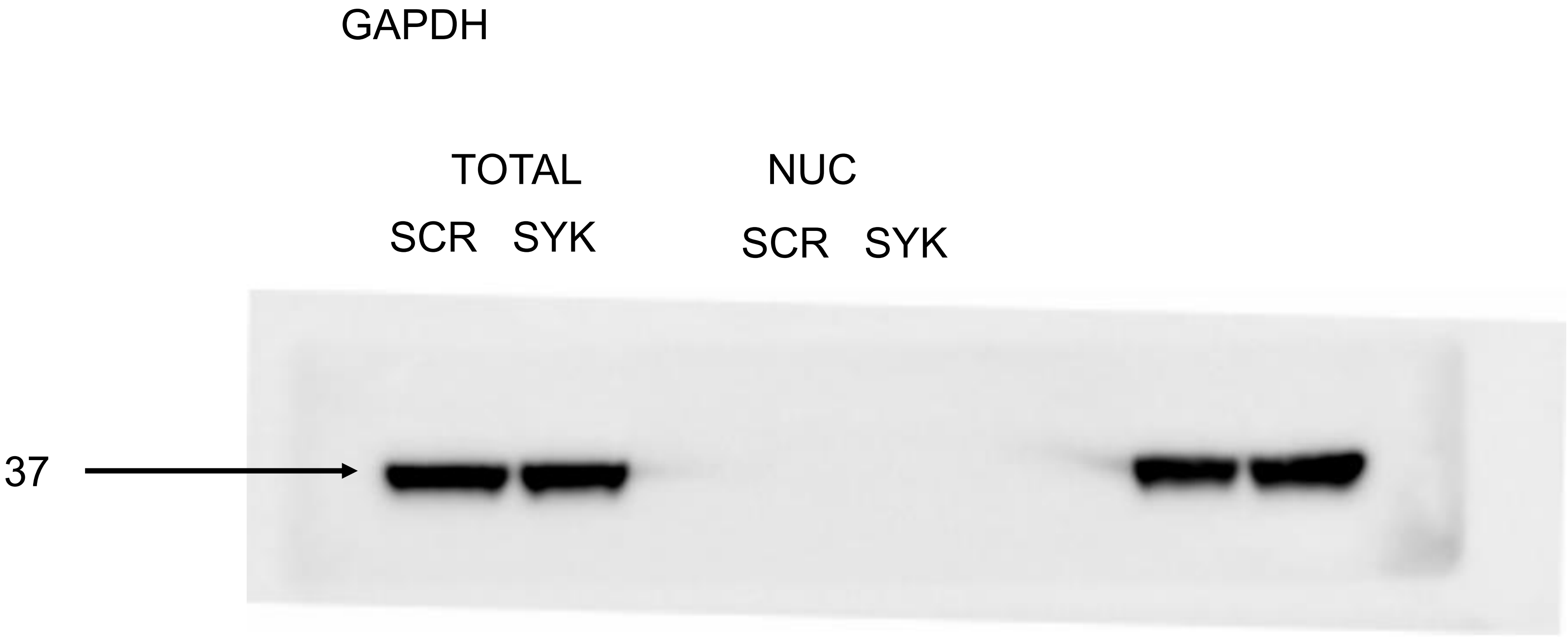

SUPP FIGURE 2D

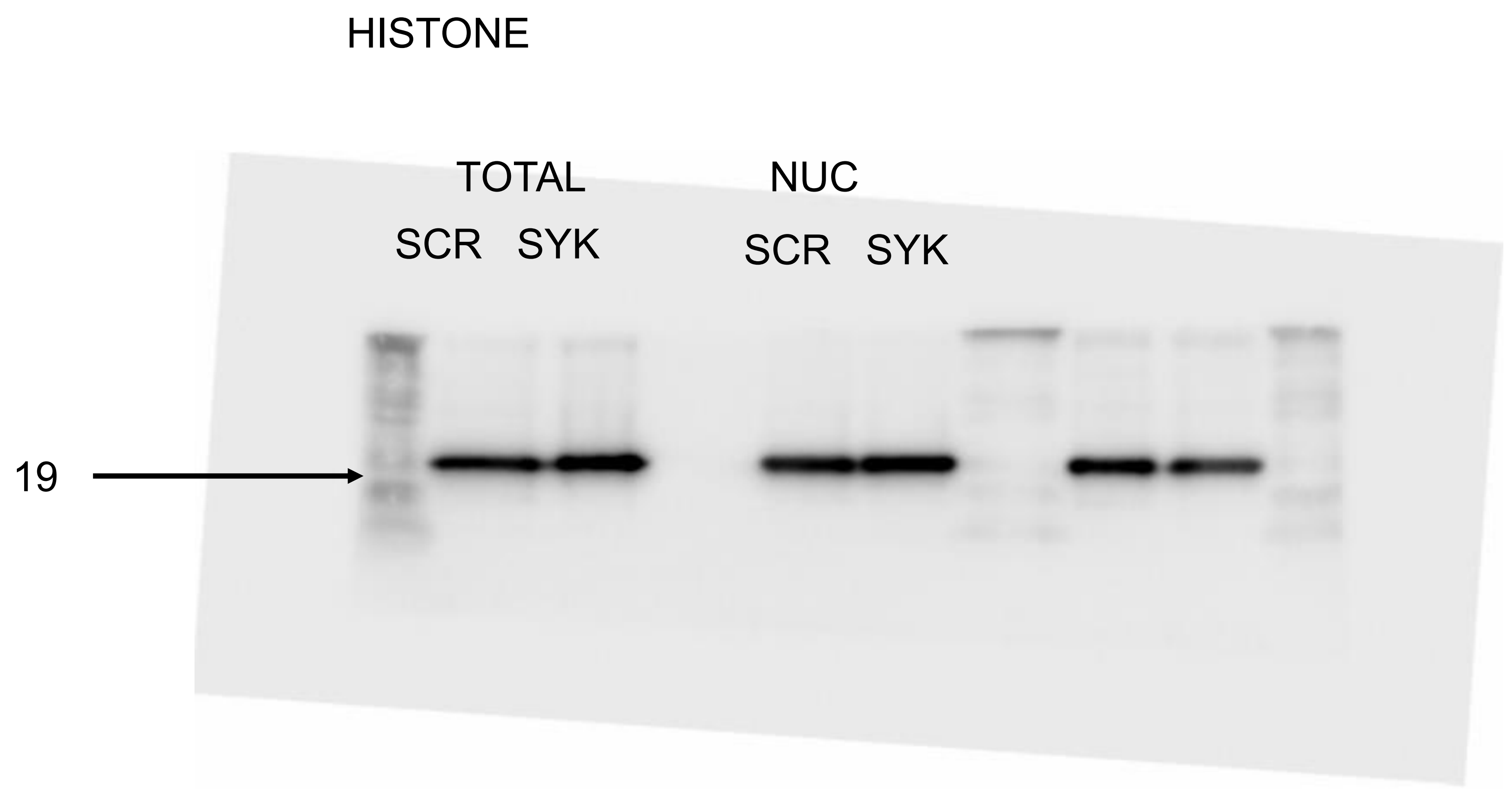

SUPP FIGURE 2D

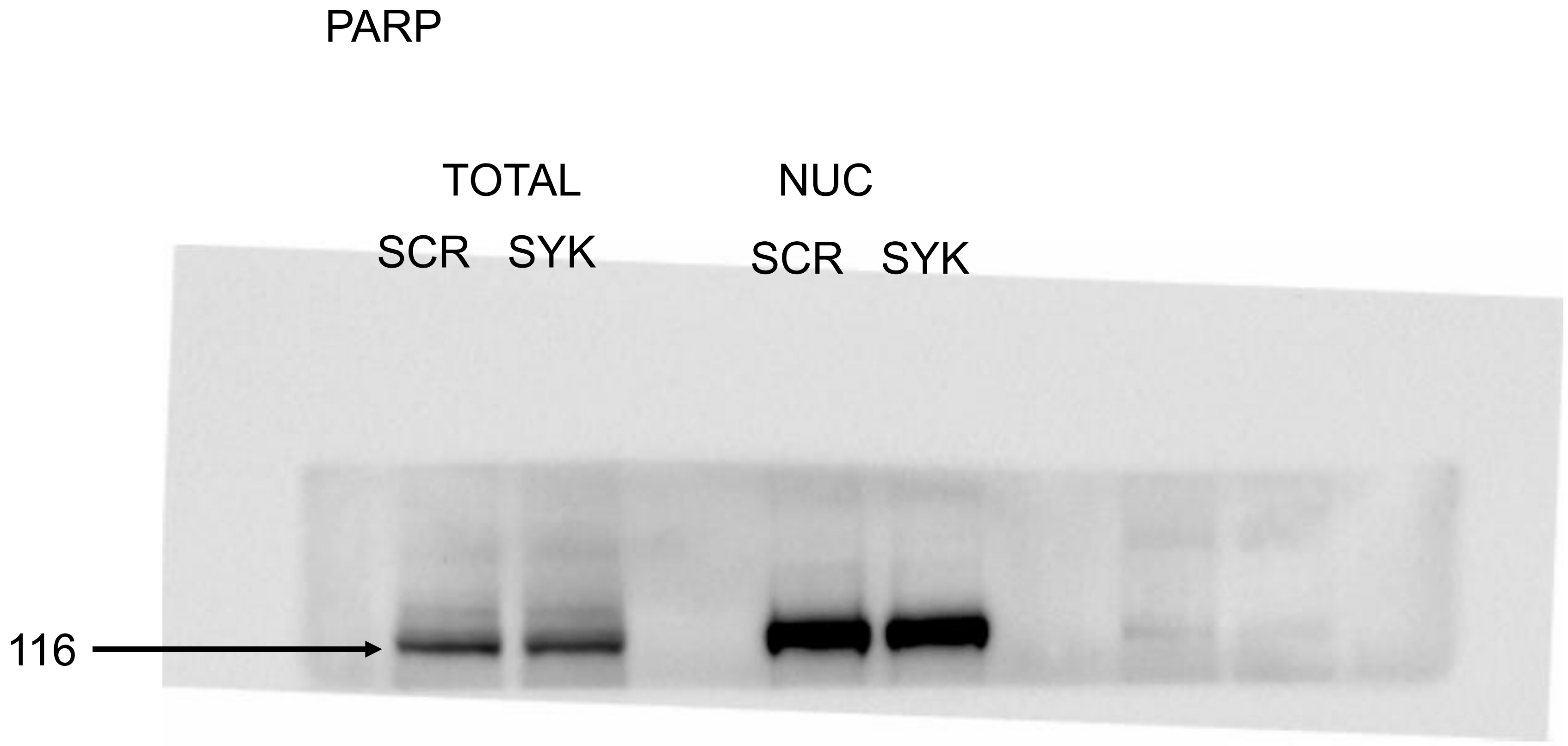

SUPP FIGURE 3B

BACTIN

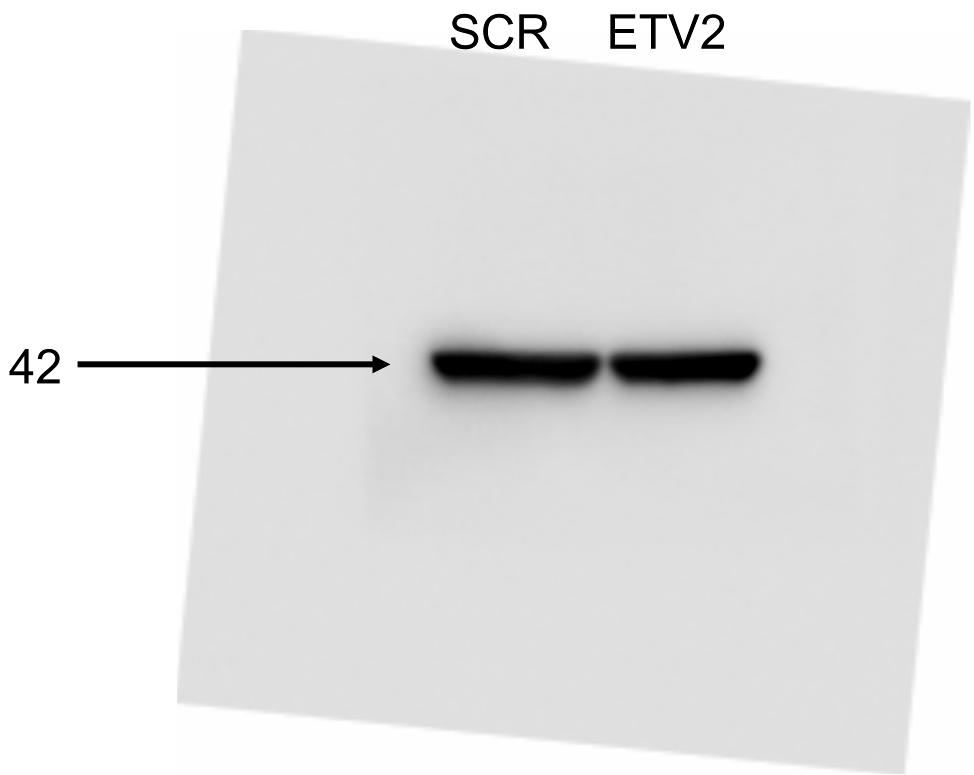

SUPP FIGURE 3B

cPARP

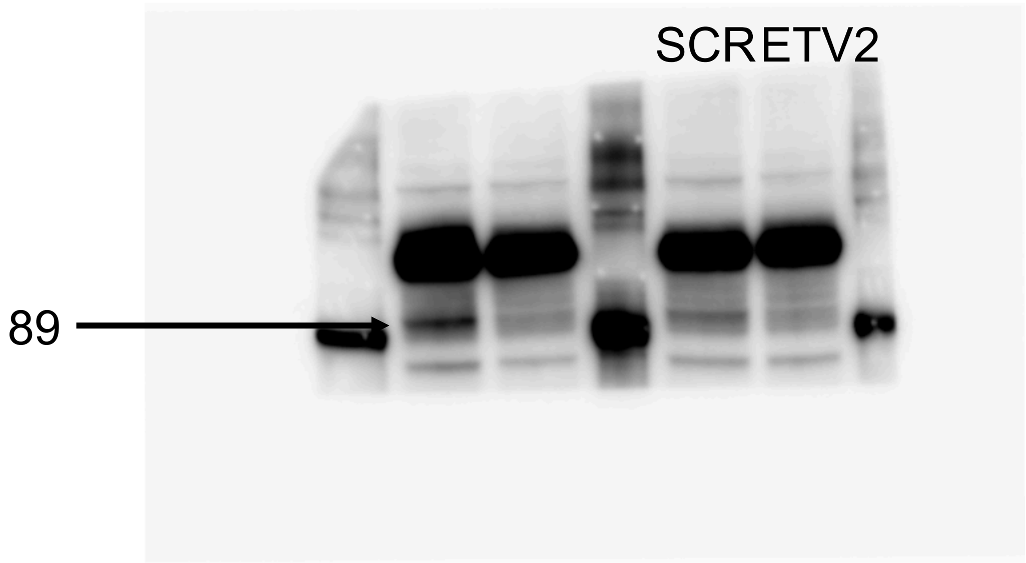

SUPP FIGURE 3B

PARP

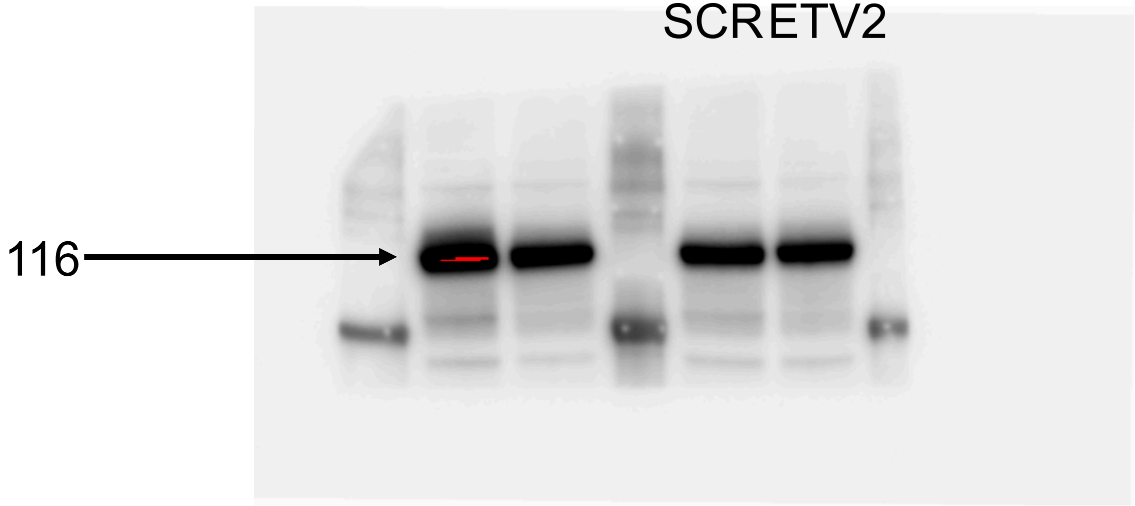

SUPP FIGURE 3B

TUBERIN

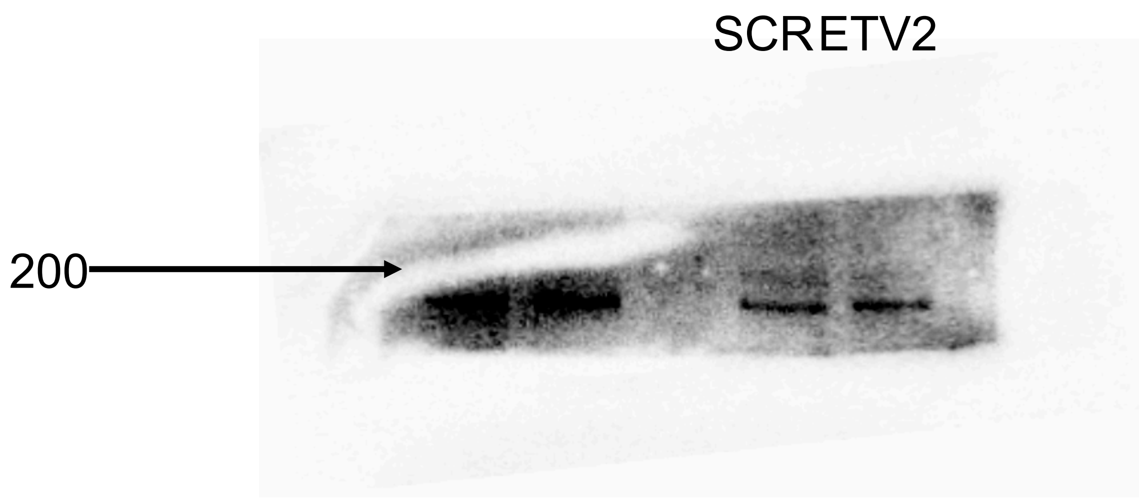

SUPP FIGURE 3B

ETV2

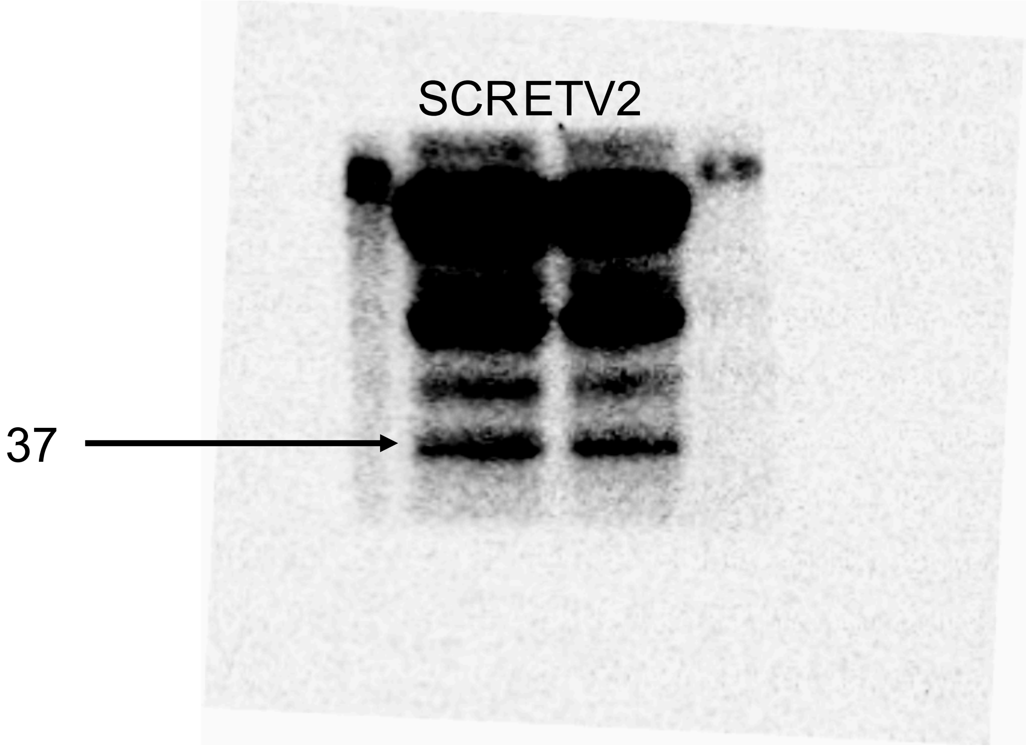

SUPP FIGURE 4B

BACTIN

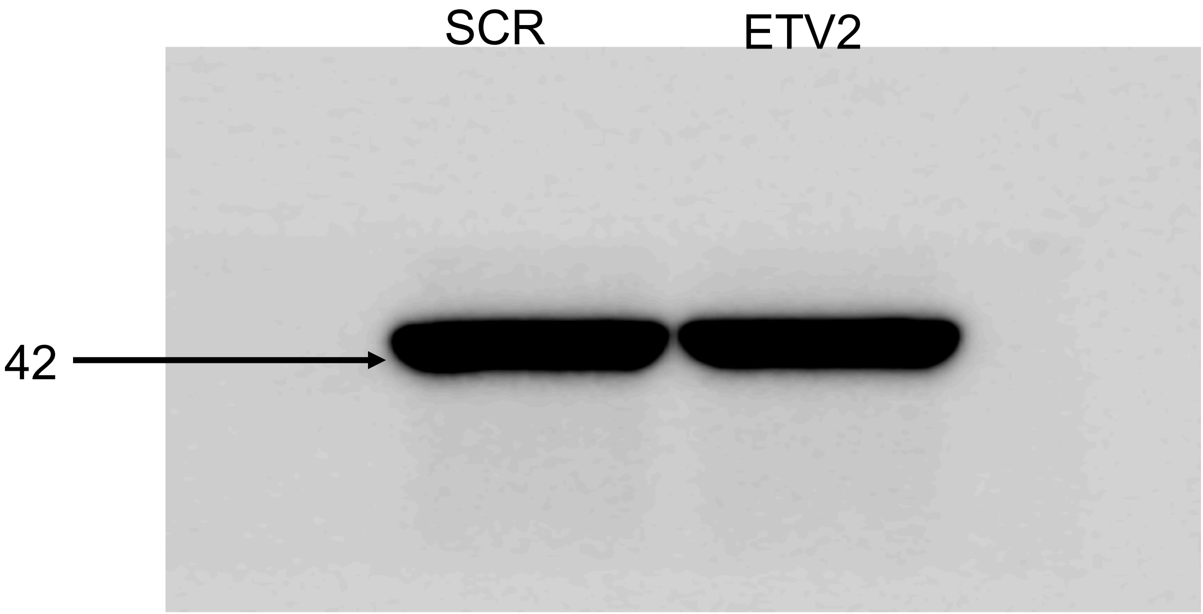

SUPP FIGURE 4B

cPARP

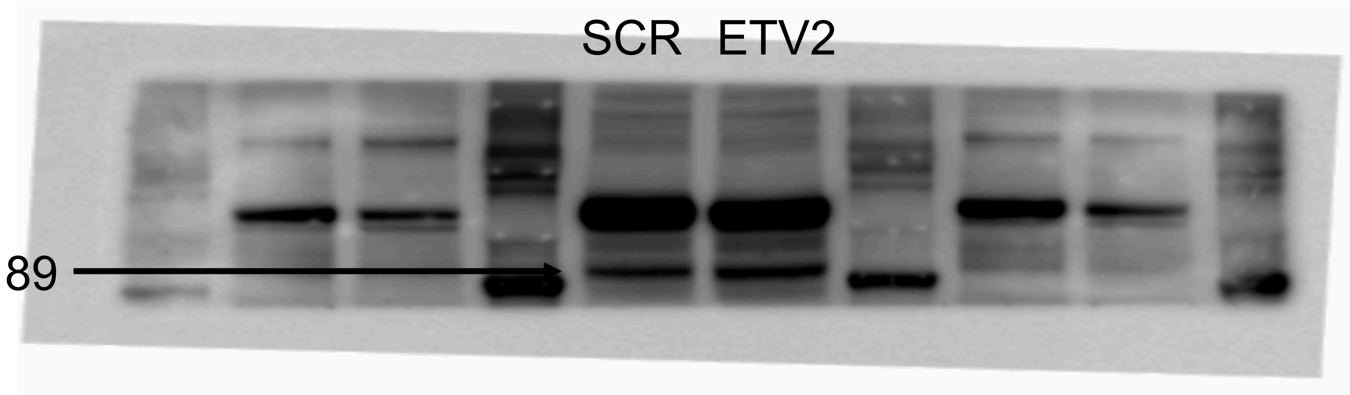

SUPP FIGURE 4B

PARP

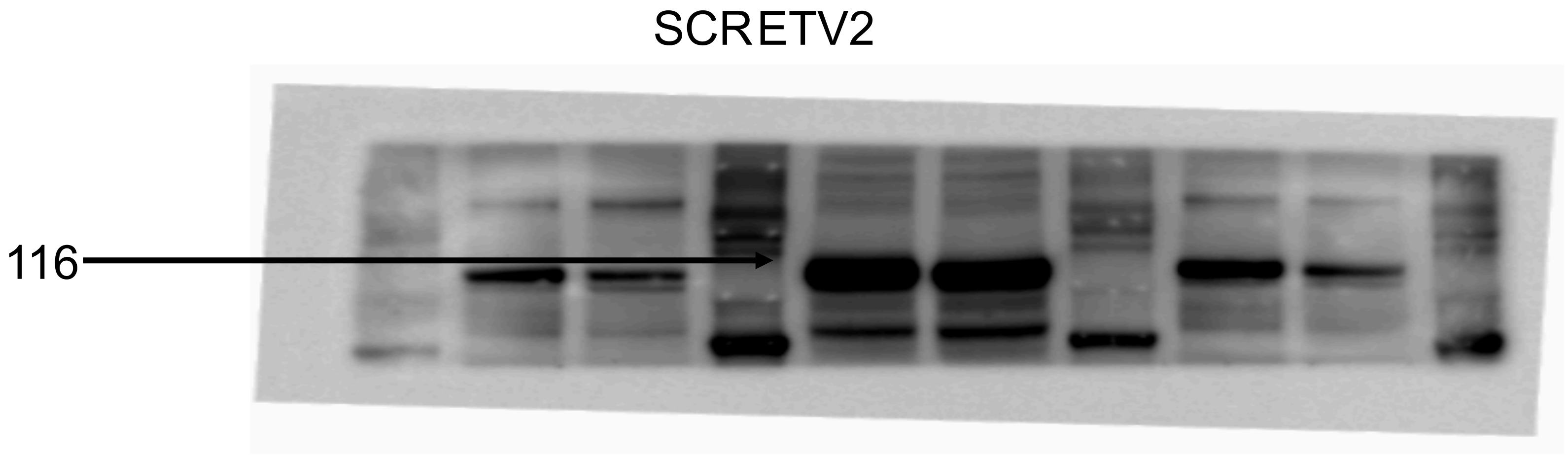

SUPP FIGURE 4B

ETV2

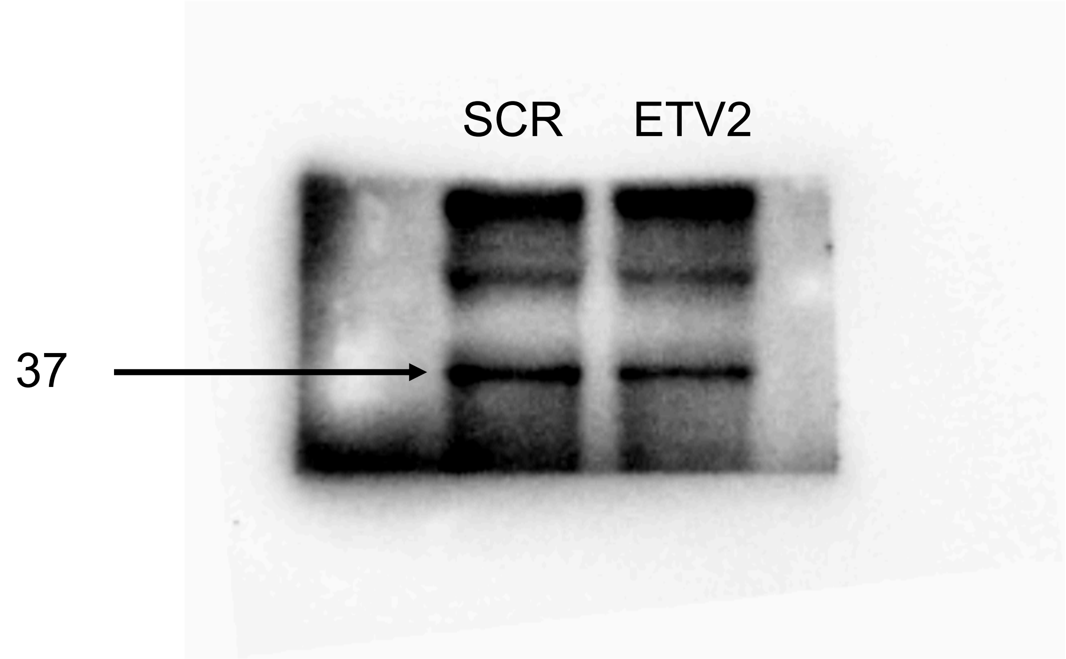

SUPP FIGURE 6

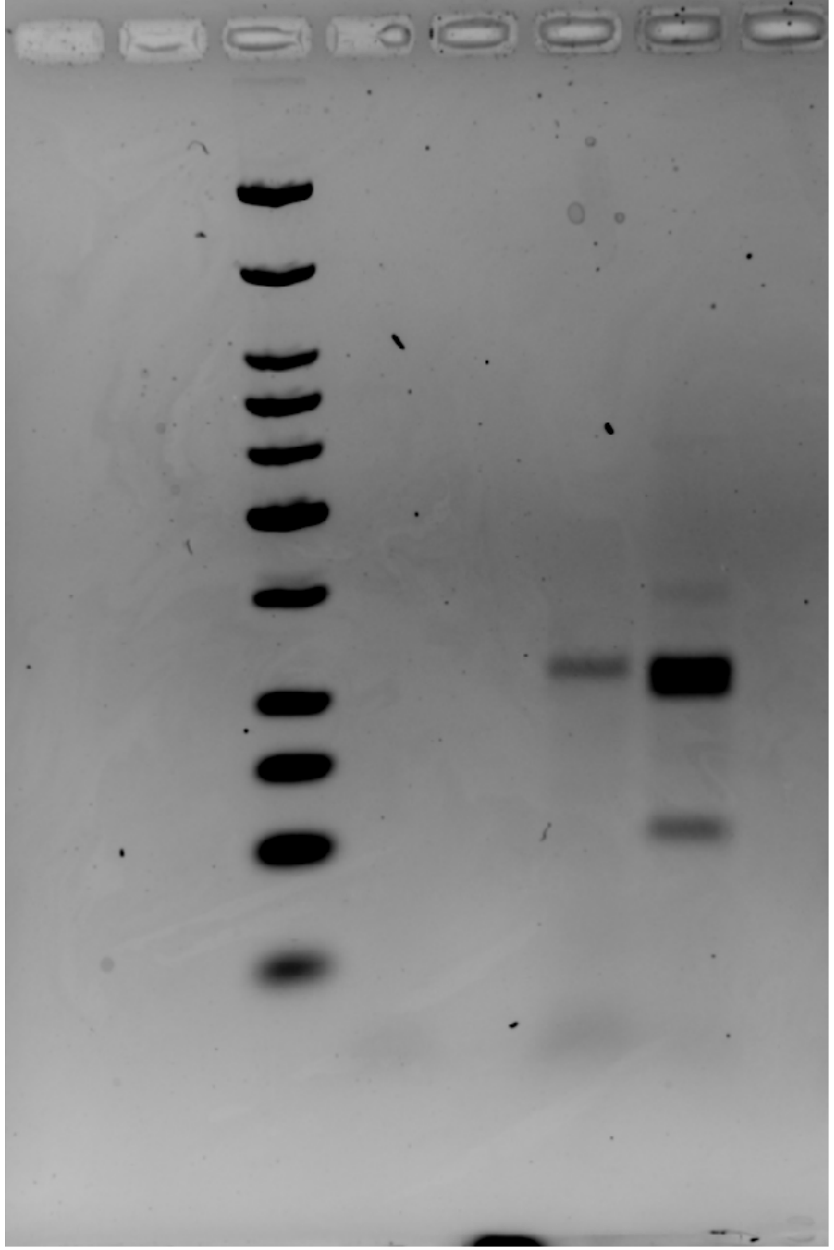

Figure 2A

DMSO	SykI	Rapamycin
0.961	0.839	1.315
0.965	0.956	1.100
1.074	1.360	1.438

DMSO v. SykI	DMSO v. Rapamycin
p = 0.8085	p = 0.0513

Figure 2B

DMSO	SykI	Rapamycin
0.709	0.683	0.671
0.578	0.444	0.579
0.978	0.979	0.641
2.186	1.445	1.588
0.549	0.572	0.782

DMSO v. SykI	DMSO v. Rapamycin
p = 0.1991	p = 0.5513

Figure 2E

DMSO	SykI
0.760	1.431
1.409	2.451
0.831	1.659

DMSO	Rapamycin
1.501	1.186
0.539	0.280
0.959	0.705

DMSO v.
SykI
p = 0.0040

DMSO v.
Rapamycin
p = 0.0905

Figure 3A

Scr siRNA	Etv2 siRNA
1.00	0.00
1.00	0.10
1.00	0.48
1.00	0.30

Scr v. Etv2
p = 0.0054

Figure 3C

Scr siRNA	Etv2 siRNA
1.00	0.45
1.00	0.30
1.00	0.60
1.00	0.80

Scr v. Etv2
p = 0.0227

Figure 3E

Scr siRNA	Etv2 siRNA
19.00	42.10
27.40	52.00
11.00	22.90

Scr v. Etv2
p = 0.0384

Figure 3G

Scr siRNA	Etv2 siRNA
1.00	2.80
1.00	3.60
1.00	1.80
1.00	2.70
1.00	1.90

Scr v. Etv2
p = 0.0091

Figure 3H

Scr siRNA	Etv2 siRNA
1.00	1.30
1.00	2.30
1.00	1.40
1.00	2.40
1.00	1.60

Scr v. Etv2
p = 0.0255

Figure 3I

Scr siRNA	Etv2 siRNA
1.00	3.40
1.00	5.20
1.00	6.00
1.00	4.00

Scr v. Etv2
p = 0.0083

Figure 3K

Scr siRNA	Etv2 siRNA
28	65
9	92
47	184
50	100
30	219
12	119
32	136
20	198
22	128
26	152
52	151
35	146
22	69
21	84
24	100
18	28
34	9
11	28
46	9

67	66
35	74
16	73
5	197
3	55
30	76
25	68
14	96
5	111
21	41
6	69
6	10
10	141
24	6
36	126
12	6
4	136
48	27
0	10
71	30
29	14
10	58
14	28
45	17
27	29
47	15
15	2
19	54
15	61
17	7
22	2
30	47
4	72
29	0
47	68
10	46
40	57
13	52
19	3
66	55
22	0
48	66
45	44
29	26
57	53

6	83
33	69
34	44
64	81
27	16
27	62
20	9
8	51
44	39
22	9
57	37
50	51
66	84
68	14
39	4
32	1
18	3
1	48
14	48
16	23
54	19
22	16
20	11
29	21
71	87
43	0
79	58
14	2
38	0
14	18
144	66
91	9
33	65
50	33
17	28
16	118
17	32
17	77
50	16
80	36
65	58
39	23
17	112
62	58
0	1

32	16
92	34
16	31
15	38
46	44
10	60
3	85
4	46
61	42
15	81
37	48
6	76
43	24
60	66
6	36
54	20
15	41
18	90
50	34
29	38
21	37
14	51
46	72
0	67
26	24
2	0
16	5
47	45
61	18
68	54
23	134
24	12
71	35
22	26
0	53
30	40
8	53
35	20
14	51
17	47
85	36
37	55
27	74
11	85
11	56

1	33
29	84
6	43
5	66
8	32
0	101
27	69
12	70
25	42
27	144
32	37
33	43
44	26
28	87
20	23
19	52
34	178
113	30
24	50
28	19
59	70
59	40
22	83
16	23
56	66
35	30
9	9
32	1
9	19
12	88
116	18
22	9
23	18
73	23
102	36
2	67
72	34
28	2
9	24
12	24
33	51
75	33
35	111
56	13
11	13

58	22
28	18
34	11
161	7
30	45
17	21
15	12
87	14
32	8
4	15
15	73
42	9
66	22
100	48
113	50
22	24
76	1
28	23
0	1
12	31
33	0
12	0

Scr v. Etv2
$p = 2.621E-06$

Figure 4A

	Scr siRNA				Etv2 siRNA			
DMSO	1.00	1.00	1.00	1.00	0.20	0.53	0.30	1.00
SykI	0.90	0.66	0.90	0.64	0.20	0.31	0.30	0.2
Rapamycin	0.40	0.27	0.60		0.20	0.17	0.20	

P-Values - T-Test

Scr siRNA: DMSO vs. SykI	0.026	
Scr siRNA: DMSO vs. Rapamycin	0.013	
Scr siRNA: SykI vs. Rapamycin	0.030	1.896E-09
Etv2 siRNA: DMSO vs. SykI	0.003	
Etv2 siRNA: DMSO vs. Rapamycin	0.001	
Etv2 siRNA: SykI vs. Rapamycin	0.153	
Scr siRNA DMSO vs. Etv2 siRNA DMSO	0.001	
Scr siRNA DMSO vs. Etv2 siRNA SykI	3.437E-07	
Scr siRNA DMSO vs. Etv2 siRNA Rapamycin	1.896E-09	
cr siRNA Rapamycin vs. Etv2 siRNA Rapamycin	0.116	

Figure 4B

						SCR siRNA						
pGL3_Basic	0.29	0.36	1.05	0.88	0.23	0.75	0.42	0.62				
pGL3_promoter-150	0.82	1.22	0.96	1.04	1.12	0.84	0.66	1.83	0.51	0.89	1.02	1.09
pGL3_promoter-386	3.42	8.09	3.15	3.39	3.54	4.66	7.14	4.5	8.56	7.08	3.34	4.04
						ETV2 siRNA						
pGL3_Basic	0.37	0.83	0.8	1.53	0.66	0.98	0.78	0.68				
pGL3_promoter-150	0.91	1.02	0.97	0.63	0.78	0.59	1.3	0.75	0.6	1.5	1.09	1.07
pGL3_promoter-386	0.27	0.25	0.59	1.03	1.88	1.18	1.25	0.64	0.65	1.38	1.64	0.99

	p-Value
pGL3_Basic	0.1309
pGL3_promoter-150	0.6048
pGL3_promoter-386	1.2982E-05

Figure 4C

Scr siRNA	'arpbp siRNA
1.00	0.20
1.00	0.01
1.00	0.01

Scr v. PARPBP
p = 0.0048

Figure 4E

Scr siRNA	'arpbp siRNA
1.00	0.60
1.00	0.40
1.00	0.40

Scr v. PARPBP
p = 0.0153

Figure 4G

Scr siRNA	'arpbp siRNA
1.00	7.60
1.00	2.70
1.00	4.20
1.00	6.10
1.00	1.50

Scr v. PARPBP
p = 0.0365

Figure 4H

Scr siRNA	'arpbp siRNA
1.00	4.30
1.00	2.20
1.00	1.50
1.00	3.50
1.00	1.80

Scr v. PARPBP
p = 0.0153

Figure 4I

Scr siRNA	'arpbp siRNA
1.00	1.36
1.00	1.48
1.00	2.09
1.00	3.26
1.00	2.82

Scr v. PARPBP
p = 0.0316

Figure 5B

	Scr siRNA			
4H	0.80	1.20	0.81	1.19
24H	0.59	1.41	1.31	0.69
48H	1.08	0.92	1.22	0.78

	ETV2 siRNA					
4H	1.57	0.79	0.67	0.72	0.81	0.43
24H	0.61	0.50	0.52	0.37	0.47	0.19
48H	0.69	0.66	0.24	0.70	0.96	0.61

Unpaired T-Test	Scr v. Etv2
4H	0.4591
24H	0.0156
48H	0.0348

Figure 5C

Scr siRNA	ETV2 siRNA
0.97	0.69
0.96	0.49
1.00	0.30
1.27	0.60
	0.59
	0.55

Figure 5D

Scr siRNA	ETV2 siRNA
0.97	0.69
0.96	0.49
1.00	0.30
1.27	0.60
	0.59
	0.55

Scr v. Etv2
p = 0.0004

Supplmentary Figure 1A

	Fabp5				Trib3				Psat1			
DMSO	1.04	1.00	0.98	0.98	1.00	1.06	0.96	0.98	0.99	1.01	0.99	1.01
Sykl	0.09	0.07	0.08	0.08	0.09	0.10	0.22	0.26	0.13	0.14	0.12	0.12
Rapa	0.06	0.06	0.06	0.07	0.05	0.01	0.13	0.12	0.10	0.03	0.09	0.09

	Tfrc				Serpina12				Loc689757			
DMSO	1.01	1.00	1.00	0.99	0.97	0.96	1.10	0.96	1.00	1.00	1.00	1.00
Sykl	0.21	0.21	0.14	0.12	0.08	0.07	0.06	0.11	0.27	0.22	0.25	0.25
Rapa	0.11	0.01	0.12	0.08	0.21	0.05	0.07	0.06	0.24	0.15	0.24	0.22

	Phgdh				Psph			
DMSO	1.09	0.96	1.00	0.96	0.88	1.10	1.15	0.86
Sykl	0.18	0.19	0.13	0.13	0.28	0.63	0.48	0.21
Rapa	0.29	0.03	0.12	0.07	0.20	0.27	0.32	0.47

Supplmentary Figure 1B

	Bub1				Ska1				Fancd2			
DMSO	1.00	1.00	1.00	1.00	0.99	0.99	1.02	0.99	1.00	1.00	1.00	1.00
Sykl	0.49	0.30	0.61	0.49	0.35	0.36	0.39	0.33	0.65	0.63	0.68	0.59
Rapa	0.35	0.04	0.22	0.25	0.23	0.18	0.25	0.27	0.30	0.01	0.37	0.33

	Arhgap11a				Kif2c				Ube2c			
DMSO	1.00	1.00	1.00	1.00	1.00	0.99	1.01	1.00	1.00	1.00	1.00	1.00
Sykl	0.53	0.67	0.55	0.50	0.46	0.43	0.48	0.37	0.55	0.54	0.48	0.46
Rapa	0.28	0.05	0.37	0.24	0.26	0.01	0.40	0.28	0.32	0.19	0.31	0.29

DMSO	Esco2				Pbk			
Sykl	1.01	0.99	1.01	0.99	0.99	1.01	1.00	1.00
Rapa	1.03	0.47	0.57	0.65	0.51	0.50	0.82	0.53
	0.43	0.23	0.31	0.44	0.32	0.18	0.47	0.39

Supplmentary Figure 1C

	Mospd2			
DMSO	1.00	1.00	1.00	1.00
Sykl	0.49	0.30	0.61	0.49
Rapa	0.35	0.04	0.22	0.25

Supplmentary Figure 1D

	Vof16				Loc100360867				Tcp11l2			
DMSO	1.07	0.98	1.00	0.95	1.02	0.99	0.99	0.99	1.00	1.00	1.00	1.00
Sykl	4.09	3.96	5.78	10.01	3.57	2.70	2.69	2.14	8.03	8.30	7.33	6.27
Rapa	3.33	2.69	5.94	8.12	2.85	0.95	4.19	2.56	8.17	1.87	9.61	7.54

	Itga8				Edn1				Col8a1			
DMSO	0.99	0.99	1.02	1.00	1.08	0.97	0.98	0.97	0.99	0.98	1.02	1.01
Sykl	4.40	9.11	3.16	1.94	2.85	4.29	3.02	7.71	5.14	7.86	5.93	3.95
Rapa	3.74	0.49	4.80	5.92	3.90	1.40	4.33	8.92	5.62	0.02	5.51	5.16

	Dusp6				Cyp3a9			
DMSO	0.98	1.02	1.00	0.99	1.01	0.99	1.01	0.99
Sykl	6.26	4.51	5.52	3.64	4.04	6.12	3.66	4.03
Rapa	6.47	1.30	5.62	7.24	4.26	1.25	3.09	10.19

Supp Figure 2A

Scr siRNA	Syk siRNA
1.00	0.06
1.00	0.15
1.00	0.41
1.00	0.04

Scr v. Syk
$p = 0.0011$

Supp Figure 2C

Scr siRNA	Syk siRNA
1.00	0.54
1.00	0.65
1.00	0.34

Scr v. Syk
$p = 0.0162$

Supp Figure 2E

Scr siRNA	Syk siRNA
1.00	1.52
1.00	1.41
1.00	0.88

Scr v. Syk
$p = 0.1528$

Supp Figure 2F

Scr siRNA	Syk siRNA
1.00	1.55
1.00	1.55
1.00	1.75

Scr v. Syk
$p = 0.0059$

Supp Figure 2G

Scr siRNA	Syk siRNA
1.00	1.80
1.00	2.06
1.00	1.81

Scr v. Syk
$p = 0.0044$

Supplementary Figure 3A

Scr siRNA	Etv2 siRNA
1.00	0.30
1.00	0.20
1.00	0.30
1.00	0.13

Scr v. Etv2
0.0002

Supplementary Figure 3C

Scr siRNA	Etv2 siRNA
1.00	0.75
1.00	0.60
1.00	0.71
1.00	0.65

Scr v. Etv2
p = 0.0014

Supplementary Figure 3D

Scr siRNA	Etv2 siRNA
1.00	1.26
1.00	1.09
1.00	0.86
1.00	1.04

Scr v. Etv2
p = 0.2510

Supplementary Figure 4A

Scr siRNA	Etv2 siRNA
1.00	0.34
1.00	0.28
1.00	0.60

Scr v. Etv2
p = 0.0132

Supplementary Figure 4C

Scr siRNA	Etv2 siRNA
1.00	0.44
1.00	0.72
1.00	0.69

Scr v. Etv2
p = 0.0255

Supplementary Figure 4D

Scr siRNA	Etv2 siRNA
1.00	1.73
1.00	2.17
1.00	1.43

Scr v. Etv2
p = 0.0344

Supplementary Figure 2A

DMSO	SykI	Rapamycin
1.00	0.58	0.34
1.00	0.62	0.03
1.00	0.78	0.46
1.00	0.55	0.35

	P-Value
DMSO vs. SykI	0.0052
DMSO vs. Rapamycin	0.0048
SykI vs. Rapamycin	0.0302

Supplementary Figure 2B

Scr siRNA	ETV2 siRNA	PARPBP siRNA
1.000	0.170	0.200
1.000	0.180	0.007
1.000	0.151	0.007

Scr siRNA vs. ETV2 siRNA	0.0001
Scr siRNA vs. PARPBP siRNA	0.0048
ETV2 siRNA vs. PARPBP siRNA	0.2703
