# Supplementary material for: ETV2 regulates PARP-1 binding protein to induce ER stress–mediated death in tuberin-deficient cells
Source: Life Sci Alliance. 2022 Feb 18;5(5):e202201369. doi: 10.26508/lsa.202201369 (PMC8860090; doi:10.26508/lsa.202201369)
Supplement: Supplementary file 2 [file LSA-2022-01369_TableS1.docx]

| **Supplementary Table 1. List of qPCR primers** | | |  |
| --- | --- | --- | --- |
|  |  |  |  |
| **Primers** | **Species** | **Sense 5' – 3'** | **Antisense 5' – 3'** |
| *Etv2* | Rat | AAACTAACCACCGAGGTC | AGCTTCTCATAATTCATTCCC |
| *Parpbp* | Rat | GGTGGAGCGTCTGTCCTTAC | GCTCCCTCAGAGACATCGTT |
| *Syk* | Rat | GAATCAAATCCTACTCCTTCC | AGCGTAAGGACTCTCATATAC |
| *Etv2* | Human | CAGCTCTCACCGTTTGCTC | AGGAACTGCCACAGCTGAAT |
| *Etv2* | Mouse | AACCGTCAGAACAAGCATCCAT | CCAAATTCCGCTCCCAGTCCT |
| Mouse–specific | Mouse | GGCATCTTCTGCTGGCTCC | GGCTAGAACCCTCCCCATTCT |
| Rat–specific | Rat | CAAGACGGATGATCAAAATGTG | TCTCTGTTTTAATCTTTGCCTCTCC |
