# Supplementary material for: ETV2 regulates PARP-1 binding protein to induce ER stress–mediated death in tuberin-deficient cells
Source: Life Sci Alliance. 2022 Feb 18;5(5):e202201369. doi: 10.26508/lsa.202201369 (PMC8860090; doi:10.26508/lsa.202201369)
Supplement: Supplementary file 3 [file LSA-2022-01369_TableS2.docx]

| **Supplementary Table 2. List of antibodies used** | |  |
| --- | --- | --- |
|  |  |  |
| **Antigen** | **Company** | **Catalog number** |
| β-ACTIN | Sigma Aldrich | A2228 |
| CD235a-PE | BD Biosciences | 561775 |
| CD44-PE | BD Biosciences | 555479 |
| CD44v6-FITC | Thermo Fischer Scientific | MA5-16966 |
| CD45-FITC | BD Biosciences | 560976 |
| CHOP | Cell Signaling | 2895 |
| p-EIF2α | Cell Signaling | 9721 |
| EIF2α | Cell Signaling | 9722 |
| ETV2 | Abcam | ab181847 |
| G3BP | Abcam | ab56574 |
| GAPDH | Cell Signaling | 2118 |
| HISTONE | Abcam | ab183902 |
| p-p70 S6 Kinase (T389) | Cell Signaling | 2711 |
| p70 S6 Kinase | Cell Signaling | 2708 |
| Cleaved-PARP (Asp214) | Cell Signaling | 9545 |
| PARP | Cell Signaling | 9542 |
| PARPBP | Origene | TA331876 |
| p-SYK (Y525/526) | Cell Signaling | 2711 |
| SYK | Cell Signaling | 13198 |
