# Supplementary material for: ETV2 regulates PARP-1 binding protein to induce ER stress–mediated death in tuberin-deficient cells
Source: Life Sci Alliance. 2022 Feb 18;5(5):e202201369. doi: 10.26508/lsa.202201369 (PMC8860090; doi:10.26508/lsa.202201369)
Supplement: Supplementary file 4 [file LSA-2022-01369_TableS3.docx]

| **Supplementary Table 3. List of siRNA** | | | | | | | | |
| --- | --- | --- | --- | --- | --- | --- | --- | --- |
|  |  | |  | |  | |  | |
| **Target gene** | | **Species** | | **Accession number** | | **Sense 5' – 3'** | **Antisense 5' – 3'** |  |
| *Etv2* | | Rat | | NC_005100 | | GUGGAAUAGUGGGCUGCAA[dT][dT] | UUGCAGCCCACUAUUCCAC[dT][dT] |  |
| *Parpbp* | | Rat | | NC_005106 | | GAAUGUUAAUUCCUCAUCA[dT][dT] | UGAUGAGGAAUUAACAUUC[dT][dT] |  |
| *Syk* | | Rat | | NC_005116 | | GGUCAAAGACCAACGGAAA[dT][dT] | UUUCCGUUGGUCUUUGACC[dT][dT] |  |
| *Etv2* | | Mouse | | NC_000073.6 | | CAUUGACUCGCUACUCCAA[dT][dT] | UUGGAGUAGCGAGUCAAUG[dT][Dt] |  |
